# Prior Authorization, Quantity Limits, and Step Therapy for Patient-Administered Antiemetics

**DOI:** 10.1001/jamanetworkopen.2025.35707

**Published:** 2025-10-06

**Authors:** Kaitlyn Vu, Arjun Gupta, Fumiko Chino, Justin Barnes, Bridgette Thom, Michael Anne Kyle

**Affiliations:** 1 Harvard College, Cambridge, Massachusetts; 2 Division of Hematology, Oncology, and Transplantation, University of Minnesota, Minneapolis; 3 Department of Radiation Oncology, MD Anderson Cancer Center, Houston, Texas; 4 Department of Radiation Oncology, Washington University School of Medicine in St Louis, St Louis, Missouri; 5 School of Social Work, University of North Carolina at Chapel Hill; 6 Department of Medical Ethics and Health Policy, Perelman School of Medicine, University of Pennsylvania, Philadelphia

## Abstract

**Importance:**

Antiemetics have level 1 evidence supporting their role in improving quality of life and treatment tolerability for patients with cancer; they are an essential part of quality care. Utilization management (prior authorization, step therapy, and quantity limits) is implemented by insurance companies to improve effectiveness and efficiency and to control costs.

**Objective:**

To characterize utilization management policies for antiemetics for Patient Protection and Affordable Care Act (ACA) Marketplace and Medicaid plans.

**Design, Setting, and Participants:**

This cross-sectional study used a national sample of all available Medicaid and ACA drug formularies in 2024 linked to the US Food and Drug Administration’s National Drug Codes. Formulations of the 13 most common self-administered (oral, sublingual, transdermal) antiemetics were identified.

**Exposure:**

Plans’ prior authorization, quantity limit, and step therapy policies.

**Main Outcomes and Measures:**

The share of antiemetics subject to utilization management by coverage type (ACA, Medicaid, Medicaid managed care, state Medicaid), generic vs brand, and geographically by state was calculated.

**Results:**

A total of 561 formularies (301 [53.7%] ACA; 260 [46.3%] Medicaid) were included, amounting to 348 215 unique drug-plan formulations (173 607 [49.9%] ACA; 174 608 [50.1%] Medicaid). Overall, utilization management affected 66 981 covered antiemetic medications in ACA plans (39.7%) and 75 727 in Medicaid plans (43.4%). In ACA plans, a greater proportion of generic formulations faced restrictions than brand-name drugs (40.5% of generic [67 931 of 167 587] vs 17.4% of brand [1050 of 6020]). In Medicaid, this was the opposite: 82.5% of brand drugs (4357 of 5280) vs 42.2% of generic drugs (71 370 of 169 328) faced restrictions. Utilization management policies varied greatly by plan and state; some plans imposed at least 1 coverage restriction on 100% of antiemetic formulations, and others none. Quantity limits were the most prevalent utilization management tool, applying to more than one-third of covered antiemetic formulations in ACA (64 198 [37.0%]) and Medicaid plans (55 585 [38.1%]).

**Conclusions and Relevance:**

In this cross-sectional study of Medicaid and ACA formularies, there was substantial variation in utilization management requirements for antiemetics across insurance type and state as well as drug type, use, and class. Efforts to increase standardization of utilization management policies may alleviate administrative burden for patients.

## Key Points

**Question:**

Are patient-administered antiemetics subject to utilization management (prior authorization, quantity limits, step therapy) in Medicaid and Patient Protection and Affordable Care Act (ACA) plan formularies?

**Findings:**

In this cross-sectional study of 561 formularies and 348 215 unique drug-plan formulations, utilization management affected 39.7% of covered antiemetic medications in ACA plans and 43.4% in Medicaid plans, with wide variation by plan, drug, and geography.

**Meaning:**

The findings of this study highlight extensive variation in patient exposure to utilization management by drug, plan, and state, underscoring the complexity patients face in attempting to secure financial coverage for supportive care medications.

## Teaser

This cross-sectional study evaluates whether antiemetic drugs for patients with cancer who have Patient Protection and Affordable Care Act (ACA) Marketplace or Medicaid plans are subject to utilization management, including prior authorization, step therapy, and quantity limits.

## Introduction

Utilization management (UM) tools, including prior authorization, step therapy, and quantity limits, are increasingly prevalent, raising concerns about timely access to care and patient outcomes.^1,2,3,4,5,6^ Physician surveys consistently report patient harm associated with UM for cancer care, including treatment delays, denials, disease progression, and even loss of life.^5,7,8^ At the same time, guideline-discordant care^9^ and financial toxicity^10,11^ are persistent challenges in cancer care, increasing pressure on administrative mechanisms such as UM to constrain utilization. Supportive care drugs are vital for managing symptoms of cancer diagnosis and treatment,^12^ but little is known about how UM policies are applied to these medications.^13,14^

Insurers use UM to influence utilization. For example, prior authorization requires prescribers to obtain preapproval for a drug or treatment for it to be covered, and quantity limits specify the amount of a medication covered in a given prescription or time frame. In recent years, UM has increased in volume and complexity,^6,15,16,17^ spurring interest in opportunities to simplify UM policies and processes.^18,19^ To date, most UM research has focused on Medicare,^1,6,16,20,21,22^ with fewer studies focused on other types of coverage (commercial,^15,17^ Patient Protection and Affordable Care Act [ACA] Marketplace plans,^23^ Medicaid^24,25^).

Cancer can induce nausea, with many patients presenting with weight loss due to vomiting and poor appetite.^26^ Chemotherapy- and radiotherapy-induced nausea and vomiting (CINV) is a prominent adverse effect of many cancer treatments, with the potential for substantial impact on patients’ quality of life and, importantly, their ability to tolerate treatment regimens.^27^ Clinical practice guidelines outline detailed protocols for CINV management, but prior research suggests antiemetics are both overused and underused relative to clinical guidelines.^9,12,28,29^ Improving guideline-concordant treatment of cancer-related symptoms is a clinical priority. As such, understanding the UM landscape for antiemetics is important: many patients receive antiemetics to take as needed for breakthrough nausea or vomiting, and coverage policies play a key role in facilitating or hindering timely access to medications.

Understanding UM policies in Medicaid and ACA Marketplace plans is important because many patients “churn” between these 2 types of coverage due to fluctuating eligibility requirements, gaps in coverage, or other factors.^30,31^ Variability in the implementation of UM requirements in both coverage types may thus affect the continuity of care for patients. Within Medicaid, states may directly administer Medicaid, contract with managed care organizations to administer coverage, or use a combination of both approaches—implications for coverage restrictions are unknown.

We sought to quantify the national landscape of UM for antiemetics to inform a more comprehensive understanding of timely access to symptom relief for patients covered by ACA or Medicaid plans. The main objectives of this study are to describe the prevalence of UM requirements for antiemetics in both markets, the differences in coverage restrictions between them, and variations in UM requirements by drug, plan, and state.

## Methods

We used 2024 Ideon formulary data for all available ACA and Medicaid plans (including Medicaid managed care and state plans), obtained through the Robert Wood Johnson Foundation’s noncommercial researcher initiative. Ideon provides data sharing services for insurance carriers, technology platforms, and researchers. We identified drugs using the US Food and Drug Administration (FDA) National Drug Codes (NDC) database and linked brand and generic drug names to the formulary files. This study was approved by the Harvard Faculty of Medicine institutional review board, and informed consent was not required given that no human participants were involved. We followed Strengthening the Reporting of Observational Studies in Epidemiology (STROBE) reporting guidelines for cross-sectional studies.^32^

We classified drugs as antiemetics using previously established methods^13^ and review of clinical guidelines.^12,33,34^ We excluded controlled substances (cannabinoids, benzodiazepines) and diversion risks (steroids) given the complexity of coverage restrictions in those groups (eAppendix in Supplement 1). We further excluded drugs that did not appear in both the ACA and Medicaid formularies. Of note, some drugs included in this study (eg, olanzapine, haloperidol, scopolamine) may have additional benefits beyond CINV prophylaxis and cancer-related nausea treatment but were included given their inclusion in guidelines for patients with cancer.^12,33,34^

The formulary files contained binary indicators for prior authorization, step therapy, and quantity limits for each drug at the NDC package code level as well as ordinal indicators for tier (eTable 7 in Supplement 1). We treated each drug-dose-plan combination as a unique observation. For example, a plan might cover several formulations of the medication ondansetron, such as ondansetron 4-mg tab (generic), ondansetron 4-mg tab (brand-name [Zofran]), ondansetron 4-mg oral disintegrating tablet, and ondansetron 4-mg injection. UM indicators for every unique drug-dose-formulation covered by each plan were captured. This level of specificity allowed us to include only routes typically filled at a retail pharmacy and self-administered at home (oral, sublingual, transdermal) and exclude routes administered in a clinical setting (intravenous). However, since we do not have utilization data, some of the included drugs may also be used in clinical settings and are not necessarily limited to patient self-administration.

### Statistical Analysis

We calculated the share of drug-dose-plan antiemetic formulations covered by each plan subject to prior authorization, step therapy, or quantity limits. For drugs with multiple doses or formulations, we aggregated to the molecule level (eg, ondansetron is a category consisting of all formulations of that drug in our sample).

We examined UM policies for covered antiemetic formulations across several dimensions: coverage type (ACA, Medicaid, Medicaid managed care, state-administered Medicaid), generic vs brand, individual drug, and geographically by state. We performed analyses using RStudio version 2024.04.2 + 764 (R Project for Statistical Computing).

## Results

### Sample Characteristics

Our dataset comprised 561 formularies, of which 301 were ACA plans (53.7%; representing 93% of the national ACA market) and 260 were Medicaid plans (46.3%; representing 90% of the national Medicaid market) (eTable 4 in Supplement 1). The Medicaid formularies consisted of 45 (17.3%) state-administered Medicaid plans and 215 (82.7%) Medicaid managed care plans. There were 403 unique NDC codes and 662 unique NDC package codes that met our inclusion criteria, representing 348 215 unique drug-plan formulations (eTable 1 in Supplement 1). Our analytic sample included 13 oral antiemetic formulations, of which 5 were brand, and 8 were generic: aprepitant (brand and generic), rolapitant (brand), netupitant/palonsetron (drug combination, brand), ondansetron (generic), haloperidol (generic), metoclopramide (brand and generic), olanzapine (brand and generic), scopolamine (generic), prochlorperazine (generic), and promethazine (generic) (eTable 2 in Supplement 1).

### UM Prevalence and Variation

In our sample, 68 981 of 173 607 unique plan-drug combinations in ACA plans (39.7%) and 75 727 of 174 608 unique plan-drug combinations in Medicaid plans (43.4%) had some form of UM. Within Medicaid, the share of antiemetics subject to some form of UM was 44.9% (63 856 of 142 170) in managed care plans and 36.6% (11 871 of 32 438) in state-administered Medicaid plans.

The share of antiemetics subject to at least 1 type of UM in ACA plans was 17.4% for brands (1050 of 6020) and 40.5% for generics (67 931 of 167 587). This is the opposite in Medicaid plans, where the share of antiemetics subject to at least 1 type of UM was 82.5% for brands (4357 of 5280) and 42.2% for generics (71 370 of 169 328). In Medicaid managed care, 82.8% of brands (3561 of 4300) and 43.7% of generics (60 295 of 137 870) were subject to UM; in state-administered Medicaid, 81.2% of brands (796 of 980) and 35.2% of generics (11 075 of 31 458) were subject to UM.

At the plan level, UM policies varied greatly. Ten ACA plans applied no UM to any of the antiemetics in our sample, while 2 applied it to virtually all (99.8%). In Medicaid, 2 plans applied no UM to any antiemetics, while 2 plans applied some form of UM to every single antiemetic (100%).

### Prior Authorization

**Figure 1.**
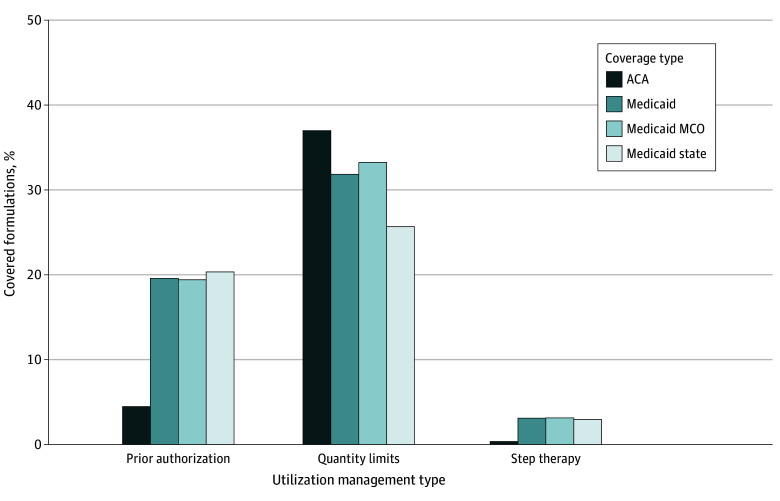
Percentage of Covered Antiemetic Formulations Subject to Utilization Management, by Coverage Type ACA indicates Patient Protection and Affordable Care Act; MCO, managed care organization.

Overall, ACA plans required prior authorization for 7761 antiemetics (4.5%), whereas Medicaid plans required prior authorization for 34 202 (19.6%) (Figure 1). Managed care and state Medicaid plans were similar, requiring prior authorization for 27 603 (19.4%) and 6599 (20.3%) antiemetics, respectively.

**Figure 2.**
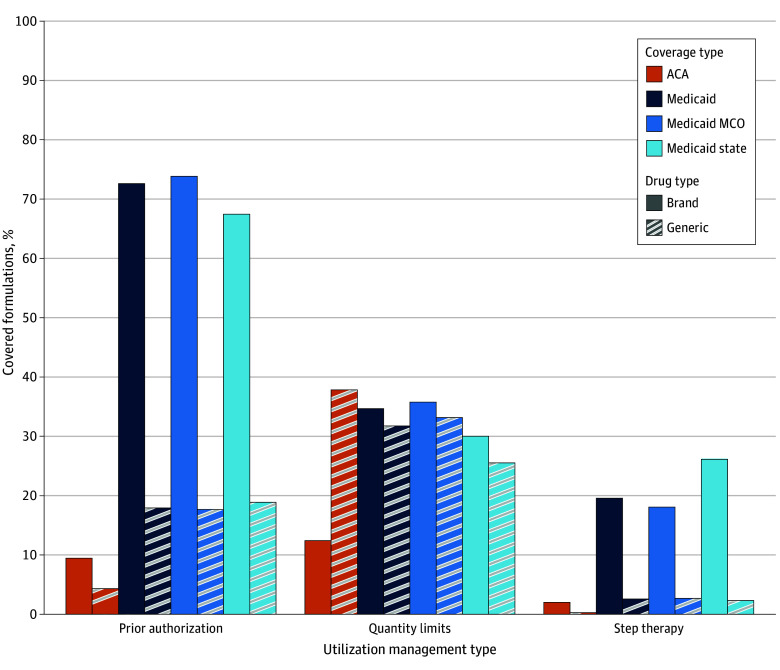
Percentage of Covered Antiemetic Formulations Subject to Utilization Management, Brand vs Generic ACA indicates Patient Protection and Affordable Care Act; MCO, managed-care organization.

Brand antiemetics were subject to prior authorization more often than generic drugs (Figure 2). In ACA plans, prior authorization was required for 571 brand antiemetics (9.5%) and 7190 generic antiemetics (4.3%). In Medicaid, prior authorization was required for 3836 brand (72.7%) and 30 366 generic (17.9%) antiemetics. Patterns were similar in both types of Medicaid coverage: in managed care, prior authorization was required for 3175 brand (73.8%) and 24 428 generic (17.7%) antiemetics; in state Medicaid, it was required for 661 brand (67.4%) and 5938 generic (18.9%) antiemetics.

**Figure 3.**
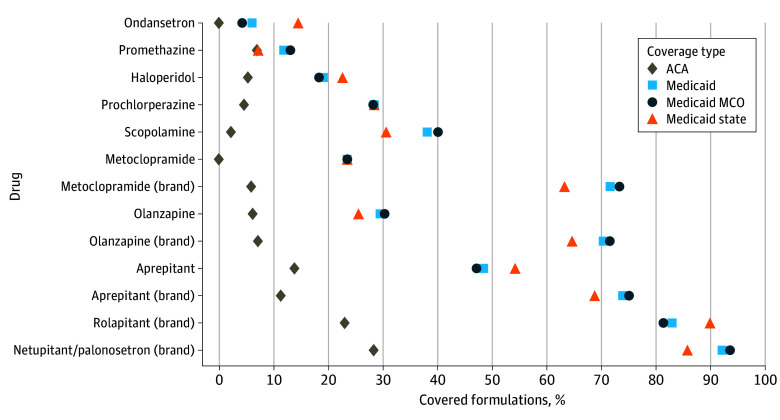
Percentage of Covered Antiemetic Formulations Subject to Prior Authorization, by Drug and Coverage Type ACA indicates Patient Protection and Affordable Care Act; MCO, managed-care organization.

There was substantial variation in prior authorization requirements by drug (Figure 3). Prior authorization was applied sparingly to ondansetron; it was not required for any ondansetron formulations in ACA plans and applied to only 3031 of 49 368 ondansetron formulations (6.1%) in Medicaid. Within Medicaid, there was variation in ondansetron prior authorization requirements in managed care (1707 of 40 205 [4.2%]) vs state-administered Medicaid (1324 of 9163 [14.4%]) plans. In contrast, covered formulations of the brand combination drug netupitant/palonosetron were extensively subject to prior authorization (ACA, 85 [28.2%]; Medicaid overall, 243 [92.0%]; Medicaid managed care, 201 [93.5%]; state-administered Medicaid, 42 [85.7%]).

Plans varied in their approach to applying prior authorization requirements. Within ACA plans, the share of antiemetics subject to prior authorization by plan was as low as zero and as high as 58.0% (eTable 5 in Supplement 1). Within Medicaid plans, the share of antiemetics subject to prior authorization ranged from 0 to 78.5% (0.6%-78.5% among managed care and 0-77.9% among state-administered plans) (eTable 6 in Supplement 1).

**Figure 4.**
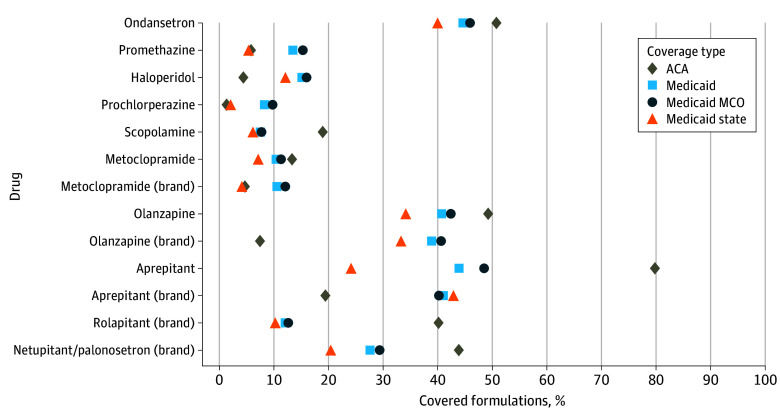
Percentage of Covered Antiemetic Formulations Subject to Quantity Limits, by Drug and Coverage Type ACA indicates Patient Protection and Affordable Care Act; MCO, managed-care organization.

Prior authorization requirements also varied by state: in ACA plans, prior authorization for antiemetics ranged from 0 in Alaska, Hawaii, North Dakota, South Dakota, and Wyoming to 27.4% in Connecticut (317 of 1158) (Figure 4). In Medicaid, prior authorization ranged from 1.7% in California (248 of 14 556) to 78.5% in Tennessee (1560 of 1986), which was the same for Medicaid managed care (eFigure 1 in Supplement 1). In state-administered Medicaid, prior authorization for antiemetics ranged from 0 in New Jersey and New Mexico to 77.9% in Arkansas (516 of 662).

### Quantity Limits

Quantity limits were the most common UM tool for the drugs in our sample (Figure 1). Quantity limits were applied to 64 198 antiemetics (37.0%) in ACA plans and 55 585 (38.1%) in Medicaid plans (managed care, 47 259 [33.2%]; state, 8326 [25.7%]).

The share of brand antiemetics subject to quantity limits was 12.4% for ACA plans (749 brand drugs) and 34.7% for Medicaid plans (overall, 1832 brand drugs; managed care, 35.8% [1528]; state, 30.0% [294]) (Figure 2). The share of generic antiemetics subject to quantity limits was 37.9% for ACA plans (63 449 generics) and 31.7% for Medicaid plans (overall, 53 753 generics; managed care, 33.2% [45 721]; state, 25.5% [8032]).

By drug, the share of antiemetics subject to quantity limits in ACA plans ranged from 1.3% for prochlorperazine (8 of 602) to 79.7% for generic aprepitant (4320 of 5418) (Figure 4). In Medicaid plans, the share of antiemetics subject to quantity limits ranged from 7.4% for scopolamine (108 of 1464) to 44.8% for ondansetron (22 139 of 49 368), with slight differences between managed care (scopolamine, 7.7% [90 of 1170]; generic aprepitant, 48.4% [1875 of 3870]) and state Medicaid (prochlorperazine, 2.0% [2 of 98]; brand aprepitant, 42.9% [63 of 147]).

Patient exposure to quantity limits varied by plan. Within ACA plans, the share of antiemetics subject to quantity limits ranged from 0 to 75.6% (eTable 5 in Supplement 1). In Medicaid, some plans did not apply quantity limits to any antiemetics, while others imposed them on 100% (managed care plans, 0-100%; state plans, 0-79.2%) (eTable 6 in Supplement 1).

**Figure 5.**
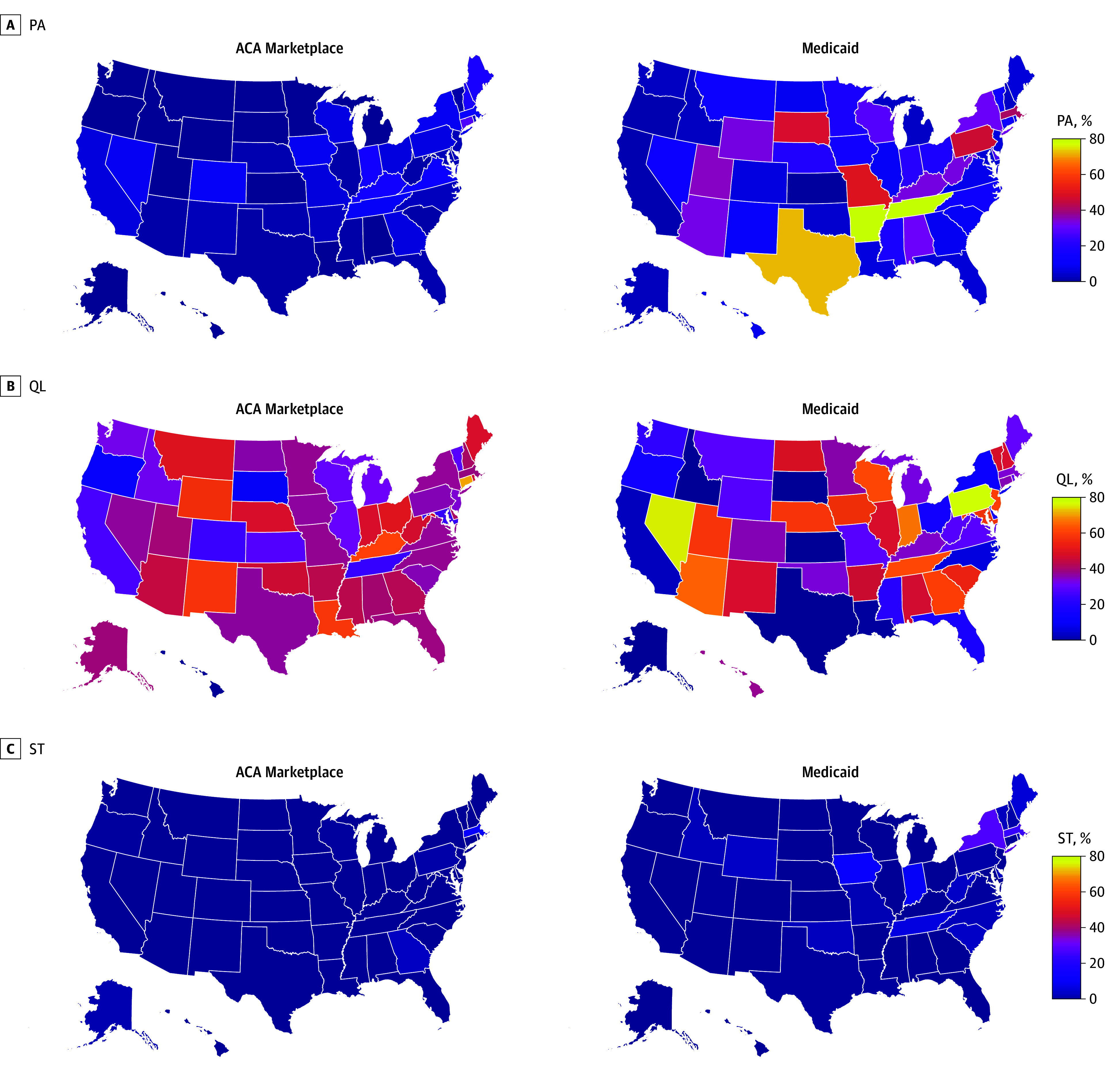
Percentage of Covered Antiemetic Formulations Subject to Utilization Management, by State and Coverage Type ACA indicates Patient Protection and Affordable Care Act; PA, prior authorization; QL, quantity limits; ST, step therapy.

Exposure to quantity limits varied by state: in ACA plans, quantity limits for antiemetics ranged from 0.3% in Hawaii (3 of 1158) to 69.6% in Connecticut (806 of 1158) (Figure 5). In Medicaid, the share of antiemetics subject to quantity limits ranged from 0 in 3 states to 74.9% in Nevada (2473 of 3302). In Medicaid managed care, the share of antiemetics subject to quantity limits ranged from 0 in Texas to 86.6% in Nevada (2285 of 2640). In state-administered Medicaid, the share of antiemetics subject to quantity limits ranged from 0 in 11 states to 79.2% in Pennsylvania (524 of 662).

### Step Therapy

Step therapy was the least common UM tool in both ACA and Medicaid formularies for the antiemetics in our sample (Figure 1). Step therapy was required for 610 antiemetics (0.4%) in ACA plans and 5423 (3.1%) in the Medicaid plans (managed care, 4462 [3.1%];state, 961 [3.0%]).

The share of brand antiemetics subject to step therapy was 2.0% for ACA plans (121) and 19.6% for Medicaid plans (overall, 1034; managed care, 18.1% [777]; state, 26.2% [257]) (Figure 2). The share of generic antiemetics subject to step therapy was 0.3% for ACA plans (489) and 2.6% for Medicaid plans (overall, 4389; managed care, 2.7% [3685]; state, 2.2% [704]).

Step therapy varied by drug (eFigure 2 in Supplement 1). No ondansetron or promethazine formulations were subject to step therapy in either ACA or Medicaid plans. Step therapy was most common for brand netupitant/palonosetron, where it was applied to 14 of 301 covered formulations in ACA plans (4.7%) and 99 in Medicaid plans (37.5%). Within Medicaid, step therapy was applied to 72 of 215 covered netupitant/palonosetron formulations in managed care (33.4%) and 27 of 49 in state-administered Medicaid (55.1%).

By plan, antiemetics subject to step therapy ranged from 0 to 36.4% within ACA plans (eTable 5 in Supplement 1). Within Medicaid plans, the share of antiemetics subject to step therapy ranged from 0 to 46.7% (managed care, 0-46.7%; state plans, 0-31.4%) (eTable 6 in Supplement 1).

Exposure to step therapy varied by state: in ACA plans, step therapy for antiemetics ranged from 0 in 20 states to 10.3% in Massachusetts (59 of 573) (Figure 5). In Medicaid, step therapy ranged from 0 in 4 states to 27.6% in New York (2558 of 9268). In Medicaid managed care, exposure to step therapy ranged from 0 in 3 states to 27.4% in New York (2361 of 8606). In state-administered Medicaid, exposure to step therapy ranged from 0 in 8 states to 31.4% in Massachusetts (208 of 662).

## Discussion

In this study, antiemetics were frequently subject to UM: we found approximately 40% of all patient-administered antiemetic formulations covered by ACA and Medicaid plans were subject to at least 1 type of coverage restriction in 2024 (eTable 3 in Supplement 1). Among drugs in our sample, quantity limits were the most common restriction. We restricted our analysis to oral, sublingual, or transdermal drug formulations to capture treatments patients are likely to bear primary responsibility for medication acquisition and administration through retail pharmacies. The implications of UM for patients likely vary by drug. For this population, the necessity of timely and effective antiemetic treatment makes administrative barriers especially concerning, as insufficient access could exacerbate symptoms.

Our findings highlight extensive variation in patient exposure to UM by drug, plan, and state. This inconsistency speaks to the complexity patients face in attempting to secure coverage for supportive care medications.

Some variation in UM by drug type is expected. For example, brand-name neurokinin-1 receptor antagonists (NK1RAs) are more expensive and typically used for planned prophylaxis—these characteristics may be compatible with restrictions such as quantity limits. However, we were surprised to see restrictions even on inexpensive, first-line rescue medications, such as all the formulations of generic ondansetron in our sample (eTable 3 in Supplement 1).

Inconsistency in coverage policies was stark in the extent of plan-level variation across all types of UM. Some plans applied quantity limits to every drug in our sample, whereas some plans did not use it at all. The magnitude of variation was smaller for prior authorization but nevertheless substantial: some plans applied prior authorization to none of the drugs in our sample, while others applied it on more than two-thirds.

While state insurance regulations and market characteristics—such as competition and plan availability—may contribute to discrepancies, we did not find an obvious explanation for the wide state-to-state variation in UM requirements for antiemetics. For example, the prevalence of UM did not align with factors such as Medicaid expansion or managed care penetration.

The higher prevalence of UM among brand antiemetics may reflect the appropriate application of coverage restrictions: they are more expensive than their generic alternatives. However, the high prevalence of coverage restrictions on antiemetics—especially for low-cost generics—is concerning, given the time-sensitive nature of nausea and vomiting. Prior authorization has been associated with delayed or discontinued care for oral anticancer medications, and similar restrictions for antiemetics could hinder timely symptom relief for patients with cancer.^2^ Nausea and vomiting are unpleasant and can reduce health-related quality of life.^27^ One of the most concerning consequences of untreated nausea is weight loss, which is associated with significantly worse overall survival.^35,36^

Differences in coverage restrictions between ACA and Medicaid plans likely reflect policy differences in program design. While both programs often serve similar patient populations, they are regulated and financed quite differently. With rare exceptions, federal law requires Medicaid to cover all FDA-approved prescription drugs, which limits Medicaid plans’ ability to exclude drugs from their formularies. As a result, Medicaid plans may be more reliant on tools like prior authorization to impose restrictions. Furthermore, beneficiary cost sharing does not play a meaningful role in Medicaid, which is financed by states and the federal government and less influenced by consumer plan selection. In contrast, ACA plans have greater discretion in designing their formularies and often operate in competitive marketplaces where consumers can compare costs and coverage before enrollment. Since beneficiary cost sharing is a prominent feature of ACA coverage, insurers may be motivated to prioritize affordability (by limiting covered treatments or applying UM, for example) to offer lower premiums that appeal to price-sensitive shoppers.^37,38^

Taken together, our findings suggest several opportunities to improve antiemetic UM policies. It may be appropriate to implement prior authorization or step therapy for costly second-line drugs; however, the rationale behind coverage restrictions in other cases is less clear. For instance, nearly one-half of plans in both the Medicaid and ACA formularies have quantity limit requirements for generic ondansetron, which is an inexpensive ($15.97 for sixty 8-mg tablets^39^ as of April 2025) and effective first-line drug.^12^

To improve timely access to care, plans and state policymakers should take a more standardized approach to designing UM policies for antiemetics. Using clinical guidelines to inform policymaking by state Medicaid programs or insurance divisions may promote more consistent, patient-centered coverage policies.

### Limitations

This study has limitations. It is descriptive and examines plan-level formulary data from a single year (2024). Additionally, this study is limited to retail antiemetics; we excluded physician-administered drugs because those formulations may be covered under medical rather than drug coverage. Our sample is incomplete, but it reflects 93% of ACA and 90% of Medicaid formularies nationally, which was all available formulary documentation. Since the UM variables are binary indicators, our data also does not contain information on the actual quantity limits of each antiemetic. Similarly, our data did not include details on coverage policies, and we cannot observe whether formularies use indication-based coverage restrictions, eg, restrictions for CINV differ from other clinical scenarios.

## Conclusions

In this cross-sectional study, we documented significant variation in UM requirements on antiemetics across the ACA and Medicaid formularies, states, drug types, uses, and classes. Efforts to increase standardization of UM policies may reduce administrative burden for patients with cancer.

## Notes

Supplement 1. **eAppendix. **Inclusion and Exclusion Criteria **eTable 1.** Sample Characteristics **eTable 2.** Antiemetic Drug Descriptions **eTable 3.** Percentage of Covered Antiemetic Formulations Subject to Utilization Management, by Formulation **eTable 4.** Number of Formulary Plans by State **eTable 5.** Percentage of Covered Antiemetic Formulations Subject to Utilization Management in ACA Marketplace, by Plan **eTable 6.** Percentage of Covered Antiemetic Formulations Subject to Utilization Management in Medicaid Marketplace, by Plan **eTable 7.** Formulary Tier Distribution for Antiemetic Drugs **eFigure 1.** Percentage of Covered Antiemetic Formulations Subject to Utilization Management, Medicaid MCO vs State **eFigure 2.** Percentage of Covered Antiemetic Formulations Subject to Step Therapy, by Drug and Coverage Type

Supplement 2.
Data Sharing Statement

## References

[1] Kyle MA, Dusetzina SB, Keating NL. Utilization management trends in Medicare Part d oncology drugs, 2010-2020. JAMA. 2023;330(3):278-280. doi:10.1001/jama.2023.10753 37462712 PMC10354675

[2] Kyle MA, Keating NL. Prior authorization and association with delayed or discontinued prescription fills. J Clin Oncol. 2024;42(8):951-960. Published online December 12, 2023. doi:10.1200/JCO.23.01693 38086013 PMC10927330

[3] Chino F, Baez A, Elkins IB, Aviki EM, Ghazal LV, Thom B. The patient experience of prior authorization for cancer care. JAMA Netw Open. 2023;6(10):e2338182. doi:10.1001/jamanetworkopen.2023.38182 37851442 PMC10585404

[4] Lin NU, Bichkoff H, Hassett MJ. Increasing burden of prior authorizations in the delivery of oncology care in the United States. J Oncol Pract. 2018;14(9):525-528. doi:10.1200/JOP.18.00428 30125129

[5] American Medical Association. 2023 AMA prior authorization physician survey. Accessed July 22, 2024. https://www.ama-assn.org/system/files/prior-authorization-survey.pdf

[6] Neprash HT, Mulcahy JF, Golberstein E. The extent and growth of prior authorization in Medicare Advantage. Am J Manag Care. 2024;30(3):e85-e92. doi:10.37765/ajmc.2024.89519 38457827

[7] ASTRO. How prior authorization harms cancer care: results of a nationwide physician survey. Accessed March 28, 2025. https://www.astro.org/ASTRO/media/ASTRO/News%20and%20Publications/PDFs/PriorAuthSurvey_2024ExecutiveSummary.pdf

[8] ASCO. ASCO 2022 prior auth survey summary. Accessed March 28, 2025. https://cdn.bfldr.com/KOIHB2Q3/as/wt2thjbxbj24ncwnwjwzvk9c/2022-ASCO-Prior-Auth-Survey-Summary

[9] Encinosa W, Davidoff AJ. Changes in antiemetic overuse in response to Choosing Wisely recommendations. JAMA Oncol. 2017;3(3):320-326. doi:10.1001/jamaoncol.2016.2530 27632203

[10] Chan RJ, Gordon LG, Tan CJ, . Relationships between financial toxicity and symptom burden in cancer survivors: a systematic review. J Pain Symptom Manage. 2019;57(3):646-660.e1. doi:10.1016/j.jpainsymman.2018.12.003 30550833

[11] Abrams HR, Durbin S, Huang CX, . Financial toxicity in cancer care: origins, impact, and solutions. Transl Behav Med. 2021;11(11):2043-2054. doi:10.1093/tbm/ibab091 34850932

[12] Hesketh PJ, Kris MG, Basch E, . Antiemetics: ASCO guideline update. J Clin Oncol. 2020;38(24):2782-2797. doi:10.1200/JCO.20.01296 32658626

[13] Gupta A, Nshuti L, Grewal US, . Financial burden of drugs prescribed for cancer-associated symptoms. JCO Oncol Pract. 2022;18(2):140-147. doi:10.1200/OP.21.00466 34558297 PMC9213200

[14] Haque W, Sedhom R, Chino F, Royce TJ, Gupta A. Payer-imposed quantity limits for antiemetics: everybody hurts. JCO Oncol Pract. 2022;18(5):313-317. doi:10.1200/OP.21.00500 34807740

[15] Lenahan KL, Nichols DE, Gertler RM, Chambers JD. Variation in use and content of prescription drug step therapy protocols, within and across health plans. Health Aff (Millwood). 2021;40(11):1749-1757. doi:10.1377/hlthaff.2021.00822 34724434

[16] Gupta R, Fein J, Newhouse JP, Schwartz AL. Comparison of prior authorization across insurers: cross sectional evidence from Medicare Advantage. BMJ. 2024;384:e077797. doi:10.1136/bmj-2023-077797 38453187 PMC10919211

[17] Jabri AZ, Asher J, Sandling J, Schulman K, Scheinker D. Variation and standardization in prior authorization requirements. medRxiv. Preprint posted online February 10, 2025. doi:10.1101/2025.02.07.25321895

[18] O’Reilly KB. Bills in 30 states show momentum to fix prior authorization. American Medical Association. May 10, 2023. Accessed July 14, 2023. https://www.ama-assn.org/practice-management/prior-authorization/bills-30-states-show-momentum-fix-prior-authorization

[19] AHIP. Health plans take action to simplify prior authorization. June 23, 2025. Accessed July 30, 2025. https://www.ahip.org/news/press-releases/health-plans-take-action-to-simplify-prior-authorization

[20] Brot-Goldberg ZC, Burn S, Layton T, Vabson B. Rationing medicine through bureaucracy: authorization restrictions in Medicare. National Bureau of Economic Research working paper 30878. Published January 2023. Accessed September 17, 2025. http://nber.org/papers/w30878

[21] Schwartz AL, Brennan TA, Verbrugge DJ, Newhouse JP. Measuring the scope of prior authorization policies: applying private insurer rules to Medicare Part B. JAMA Health Forum. 2021;2(5):e210859. doi:10.1001/jamahealthforum.2021.0859 35977311 PMC8796979

[22] Schwartz AL, Chen Y, Jagmin CL, . Coverage denials: government and private insurer policies for medical necessity in Medicare. Health Aff (Millwood). 2022;41(1):120-128. doi:10.1377/hlthaff.2021.01054 34982629 PMC9465897

[23] McManus KA, Powers S, Killelea A, Tello-Trillo S, Rogawski McQuade E. Regional disparities in qualified health plans’ prior authorization requirements for HIV pre-exposure prophylaxis in the United States. JAMA Netw Open. 2020;3(6):e207445. doi:10.1001/jamanetworkopen.2020.7445 32492164 PMC7272119

[24] Burn S, Ristovska L. Informative ordeals in healthcare: Prior authorization of drugs in Medicaid. Paper presented at: Economic Analysis of Regulation; April 26, 2024. Cambridge, Massachusetts.

[25] Grimm CA. High rates of prior authorization denials by some plans and limited state oversight raise concerns about access to care in Medicaid managed care. US Department of Health and Human Services, Office of Inspector General. July 17, 2023. Accessed August 27, 2025. https://oig.hhs.gov/reports/all/2023/high-rates-of-prior-authorization-denials-by-some-plans-and-limited-state-oversight-raise-concerns-about-access-to-care-in-medicaid-managed-care/

[26] Keeley PW. Nausea and vomiting in people with cancer and other chronic diseases. BMJ Clin Evid. 2009;2009:2406. 19445763 PMC2907825

[27] Sommariva S, Pongiglione B, Tarricone R. Impact of chemotherapy-induced nausea and vomiting on health-related quality of life and resource utilization: a systematic review. Crit Rev Oncol Hematol. 2016;99:13-36. doi:10.1016/j.critrevonc.2015.12.001 26697988

[28] Check DK, Basch EM. Appropriate use of antiemetics to prevent chemotherapy-induced nausea and vomiting. JAMA Oncol. 2017;3(3):307-309. doi:10.1001/jamaoncol.2016.2616 27631790 PMC5558447

[29] Mahendraratnam N, Farley JF, Basch E, Proctor A, Wheeler SB, Dusetzina SB. Characterizing and assessing antiemetic underuse in patients initiating highly emetogenic chemotherapy. Support Care Cancer. 2019;27(12):4525-4534. doi:10.1007/s00520-019-04730-3 30915567

[30] Wolf E, Slosar M, Menashe I. Assessment of churn in coverage among California’s health insurance marketplace enrollees. JAMA Health Forum. 2022;3(12):e224484. doi:10.1001/jamahealthforum.2022.4484 36459160 PMC9719048

[31] Shafer PR, Hinde JM. Medicaid applications spike during marketplace open enrollment: lessons from covered California. J Health Care Poor Underserved. 2022;33(3):1155-1162. doi:10.1353/hpu.2022.0102 36245153 PMC13576524

[32] Vandenbroucke JP, von Elm E, Altman DG, ; STROBE initiative. Strengthening the Reporting of Observational Studies in Epidemiology (STROBE): explanation and elaboration. Ann Intern Med. 2007;147(8):W163-94. doi:10.7326/0003-4819-147-8-200710160-00010-w1 17938389

[33] National Comprehensive Cancer Network. NCCN guidelines: supportive care. NCCN. Accessed February 13, 2022. https://www.nccn.org/guidelines/category_3

[34] National Comprehensive Cancer Network. NCCN guidelines for patients: nausea and vomiting. Accessed August 27, 2025. https://www.nccn.org/patients/guidelines/content/PDF/nausea-patient.pdf

[35] Shang L, Hattori M, Fleming G, . Impact of post-diagnosis weight change on survival outcomes in Black and White breast cancer patients. Breast Cancer Res. 2021;23(1):18. doi:10.1186/s13058-021-01397-9 33541403 PMC7863526

[36] Yuan Q, Du M, Loehrer E, . Postdiagnosis BMI change is associated with non-small cell lung cancer survival. Cancer Epidemiol Biomarkers Prev. 2022;31(1):262-268. doi:10.1158/1055-9965.EPI-21-0503 34728470 PMC8755617

[37] Tebaldi P. Estimating equilibrium in health insurance exchanges: price competition and subsidy design under the ACA. Rev Econ Stud. 2025;92(1):586-620. doi:10.1093/restud/rdae020

[38] Chu RC, Rudich J, Lee A, Peters C, Lew ND, Sommers BD. Facilitating consumer choice: standardized plans in health insurance marketplaces. Office of the Assistant Secretary for Planning and Evaluation. December 28, 2021. Accessed August 27, 2025. https://aspe.hhs.gov/reports/standardized-plans-health-insurance-marketplaces

[39] CostPlus. Ondansetron (generic for Zofran ODT). Accessed August 27, 2025. https://www.costplusdrugs.com/medications/ondansetron-8mg-orallydisintegratingtablet-odt/

