# Supplementary material for: Prior Authorization, Quantity Limits, and Step Therapy for Patient-Administered Antiemetics
Source: JAMA Netw Open. 2025 Oct 6;8(10):e2535707. doi: 10.1001/jamanetworkopen.2025.35707 (PMC12501812; doi:10.1001/jamanetworkopen.2025.35707)
Supplement: Supplement 1. — eAppendix. Inclusion and Exclusion Criteria eTable 1. Sample Characteristics eTable 2. Antiemetic Drug Descriptions eTable 3. Percentage of Covered Antiemetic Formulations Subject to Utilization Management, by Formulation eTable 4. Number of Formulary Plans by State eTable 5. Percentage of Covered Antiemetic Formulations Subject to Utilization Management in ACA Marketplace, by Plan eTable 6. Percentage of Covered Antiemetic Formulations Subject to Utilization Management in Medicaid Marketplace, by Plan eTable 7. Formulary Tier Distribution for Antiemetic Drugs eFigure 1. Percentage of Covered Antiemetic Formulations Subject to Utilization Management, Medicaid MCO vs State eFigure 2. Percentage of Covered Antiemetic Formulations Subject to Step Therapy, by Drug and Coverage Type [file jamanetwopen-e2535707-s001.pdf]

# Supplemental Online Content

Vu K, Gupta A, Chino F, Barnes J, Thom B, Kyle MA. Prior authorization, quantity limits, and step therapy for patient-administered antiemetics. *JAMA Netw Open*. 2025;8(10):e2535707. doi:10.1001/jamanetworkopen.2025.35707

**eAppendix.** Inclusion and Exclusion Criteria

**eTable 1.** Sample Characteristics

**eTable 2.** Antiemetic Drug Descriptions

**eTable 3.** Percentage of Covered Antiemetic Formulations Subject to Utilization Management, by Formulation

**eTable 4.** Number of Formulary Plans by State

**eTable 5.** Percentage of Covered Antiemetic Formulations Subject to Utilization Management in ACA Marketplace, by Plan

**eTable 6.** Percentage of Covered Antiemetic Formulations Subject to Utilization Management in Medicaid Marketplace, by Plan

**eTable 7.** Formulary Tier Distribution for Antiemetic Drugs

**eFigure 1.** Percentage of Covered Antiemetic Formulations Subject to Utilization Management, Medicaid MCO vs State

**eFigure 2.** Percentage of Covered Antiemetic Formulations Subject to Step Therapy, by Drug and Coverage Type

This supplemental material has been provided by the authors to give readers additional information about their work.

© 2025 Vu K et al. *JAMA Network Open*.

**eAppendix. Inclusion and Exclusion Criteria**

**Drugs used to treat CINV per Gupta et al.**
aprepitant capsule (generic), aprepitant capsule (Emend), rolapitant tablet (Varubi), netupitant/palonosetron capsule (Akynzeo), granisetron tablet (generic), granisetron patch (Sancuso), ondansetron tablet (generic), ondansetron tablet (Zofran), ondansetron ODT (generic), ondansetron ODT (Zuplenz), dolasetron tablet (Anzemet), dexamethasone tablet (generic), olanzapine tablet (generic), olanzapine tablet (Zyprexa), olanzapine ODT (generic), olanzapine ODT (Zyprexa Zydis), metoclopramide tablet (generic), metoclopramide tablet (Reglan), prochlorperazine tablet (generic), prochlorperazine suppository (generic), scopolamine patch (generic), promethazine tablet (generic), promethazine syrup (generic), promethazine suppository (generic), haloperidol tablet (generic), dronabinol capsule (generic), dronabinol capsule (Marinol), dronabinol oral solution (Syndros), Nabilone capsule (Cesamet), lorazepam tablet (generic), lorazepam tablet (Ativan)

**Excluded, controlled or risk for diversion**
dexamethasone tablet (generic), dronabinol capsule (generic), dronabinol capsule (Marinol), dronabinol oral solution (Syndros), Nabilone capsule (Cesamet), lorazepam tablet (generic), lorazepam tablet (Ativan)

**Excluded, not present in both ACA and Medicaid formularies**
granisetron tablet (generic), granisetron patch (Sancuso), ondansetron ODT (Zuplenz), dolasetron tablet (Anzemet)

© 2025 Vu K et al. *JAMA Network Open*.

**eTable 1.** Sample Characteristics

| | ACA | Medicaid | Medicaid MCO | Medicaid State |
|---|---|---|---|---|
| Plans | 301 | 260 | 215 | 45 |
| NDC Drug Codes | 354 | 403 | 403 | 403 |
| NDC Package Codes | 579 | 662 | 662 | 662 |
| Total Plan*Drug Combinations | 173607 | 174608 | 142170 | 32438 |
| Aprepitant | 5418 | 4752 | 3870 | 882 |
| Aprepitant (Emend) | 903 | 792 | 645 | 147 |
| Haloperidol | 32207 | 27456 | 22360 | 5096 |
| Metoclopramide | 301 | 1016 | 820 | 196 |
| Metoclopramide (Reglan) | 602 | 528 | 430 | 98 |
| Netupitant/Palonosetron (Akynzeo) | 301 | 264 | 215 | 49 |
| Olanzapine | 56287 | 49896 | 40635 | 9261 |
| Olanzapine (Zyprexa) | 3913 | 3432 | 2795 | 637 |
| Ondansetron | 56287 | 49368 | 40205 | 9163 |
| Prochlorperazine | 602 | 528 | 430 | 98 |
| Promethazine | 14749 | 34848 | 28380 | 6468 |
| Rolapitant (Varubi) | 301 | 264 | 215 | 49 |
| Scopolamine | 1134 | 1464 | 1170 | 294 |
| Brand | 6020 | 5280 | 4300 | 980 |
| Generic | 167587 | 169328 | 137870 | 31438 |

© 2025 Vu K et al. *JAMA Network Open*.

**eTable 2.** Antiemetic Drug Descriptions

| Generic Name | Brand Name | Generic Y/N | Prophylaxis | Treatment | Class |
|---|---|---|---|---|---|
| Aprepitant | Emend | 0 | 1 | 0 | NK1RA |
| Aprepitant | Aprepitant | 1 | 1 | 0 | NK1RA |
| Haloperidol | Haloperidol | 1 | 0 | 1 | Miscellaneous |
| Metoclopramide | Reglan | 0 | 0 | 1 | Miscellaneous |
| Metoclopramide | Metoclopramide | 1 | 0 | 1 | Miscellaneous |
| Netupitant and Palonosetron | Akynzeo | 0 | 1 | 0 | Miscellaneous |
| Olanzapine | Zyprexa | 0 | 1 | 1 | Miscellaneous |
| Olanzapine | Olanzapine | 1 | 1 | 1 | Miscellaneous |
| Ondansetron | Ondansetron | 1 | 1 | 1 | 5HT3RA |
| Prochlorperazine | Prochlorperazine | 1 | 0 | 1 | Miscellaneous |
| Promethazine | Promethazine | 1 | 0 | 1 | Miscellaneous |
| Rolapitant | Varubi | 0 | 1 | 0 | NK1RA |
| Scopolamine | Scopolamine | 1 | 0 | 1 | Miscellaneous |

© 2025 Vu K et al. *JAMA Network Open*.

**eTable 3.** Percentage of Covered Antiemetic Formulations Subject to Utilization Management, by Formulation

| | | | ACA | | | Medicaid | | | Medicaid MCO | | | Medicaid State | | |
|---|---|---|---|---|---|---|---|---|---|---|---|---|---|---|
| **Drug Name** | **Generic Y/N** | **Formulations** | **PA (%)** | **ST (%)** | **QL (%)** | **PA (%)** | **ST (%)** | **QL (%)** | **PA (%)** | **ST (%)** | **QL (%)** | **PA (%)** | **ST (%)** | **QL (%)** |
| Aprepitant | 1 | capsule | 13.7 | 2.2 | 81.5 | 48.5 | 4.5 | 44.4 | 47.3 | 3.3 | 48.9 | 53.5 | 10.2 | 24.5 |
| Aprepitant | 1 | kit (oral) | 14.3 | 2.3 | 70.8 | 48.5 | 9.1 | 41.7 | 46.5 | 7.9 | 46.0 | 57.1 | 14.3 | 22.4 |
| Aprepitant, Emend | 0 | capsule | 10.3 | 3.7 | 9.6 | 66.3 | 21.2 | 50.0 | 66.5 | 19.5 | 49.3 | 65.3 | 28.6 | 53.1 |
| Aprepitant, Emend | 0 | kit (oral) | 9.3 | 2.3 | 11.6 | 68.9 | 20.8 | 50.0 | 70.2 | 21.4 | 48.4 | 63.3 | 18.4 | 57.1 |
| Aprepitant, Emend | 0 | power, for suspension (oral) | 14.3 | 2.0 | 36.9 | 86.4 | 23.1 | 22.7 | 88.4 | 20.9 | 23.7 | 77.6 | 32.7 | 18.4 |
| Haloperidol | 1 | solution | 5.3 | 0.0 | 5.0 | 20.5 | 0.8 | 11.0 | 19.5 | 0.5 | 11.2 | 24.5 | 2.0 | 10.2 |
| Haloperidol | 1 | tablet | 5.3 | 0.0 | 2.0 | 19.0 | 0.4 | 15.5 | 18.2 | 0.5 | 16.3 | 22.4 | 0.0 | 12.2 |
| Metoclopramide | 1 | solution | 0.0 | 0.3 | 13.3 | 4.2 | 0.0 | 12.5 | 3.7 | 0.0 | 13.5 | 6.1 | 0.0 | 8.2 |
| Metoclopramide | 1 | oral disintegrating tablet | NA | NA | NA | 44.3 | 5.5 | 8.4 | 45.1 | 2.8 | 9.0 | 40.8 | 16.3 | 6.1 |
| Metoclopramide, Reglan | 0 | tablet | 6.0 | 0.0 | 4.7 | 71.4 | 4.9 | 10.6 | 73.3 | 2.8 | 12.1 | 63.3 | 14.3 | 4.1 |
| Netupitant and Palonosetron, Akynzeo | 0 | capsule | 28.2 | 4.7 | 43.9 | 92.0 | 37.5 | 27.7 | 93.5 | 33.5 | 29.3 | 85.7 | 55.1 | 20.4 |
| Olanzapine | 1 | tablet | 6.3 | 0.3 | 52.9 | 23.8 | 7.2 | 42.8 | 24.6 | 7.9 | 44.6 | 20.4 | 4.1 | 34.7 |
| Olanzapine | 1 | film coated tablet | 6.3 | 0.3 | 53.0 | 23.8 | 7.2 | 42.8 | 24.6 | 7.9 | 44.6 | 20.4 | 4.1 | 34.7 |
| Olanzapine | 1 | oral disintegrating tablet | 6.6 | 1.3 | 40.9 | 41.8 | 9.5 | 36.7 | 43.0 | 9.3 | 37.6 | 36.7 | 10.2 | 32.8 |
| Olanzapine, Zyprexa | 0 | kit (oral) | 11.6 | 8.0 | 15.8 | 62.1 | 35.2 | 31.4 | 64.2 | 35.8 | 33.5 | 53.1 | 32.7 | 22.4 |
| Olanzapine, Zyprexa | 0 | tablet | 5.6 | 0.0 | 4.5 | 75.3 | 17.4 | 44.4 | 76.6 | 16.3 | 45.8 | 69.4 | 22.4 | 38.1 |
| Olanzapine, Zyprexa | 0 | oral disintegrating tablet | 6.0 | 0.0 | 5.6 | 68.9 | 9.1 | 37.4 | 69.5 | 7.0 | 38.1 | 66.3 | 18.4 | 34.2 |
| Ondansetron | 1 | solution | 0.0 | 0.0 | 56.1 | 10.6 | 0.0 | 37.5 | 9.3 | 0.0 | 38.1 | 16.3 | 0.0 | 34.7 |
| Ondansetron | 1 | tablet | 0.0 | 0.0 | 53.3 | 6.1 | 0.0 | 44.9 | 4.2 | 0.0 | 46.3 | 14.3 | 0.0 | 38.8 |
| Ondansetron | 1 | film coated tablet | 0.0 | 0.0 | 53.3 | 6.1 | 0.0 | 44.9 | 4.2 | 0.0 | 46.3 | 14.3 | 0.0 | 38.8 |
| Ondansetron | 1 | oral disintegrating tablet | 0.0 | 0.0 | 49.6 | 5.7 | 0.0 | 45.6 | 3.7 | 0.0 | 46.7 | 14.3 | 0.0 | 40.8 |
| Prochlorperazine | 1 | suppository | 4.7 | 0.3 | 1.3 | 28.4 | 2.3 | 8.3 | 28.4 | 1.4 | 9.8 | 28.6 | 6.1 | 2.0 |
| Promethazine | 1 | solution | 7.0 | 0.0 | 6.6 | 13.3 | 0.0 | 14.8 | 14.0 | 0.0 | 17.7 | 10.2 | 0.0 | 2.0 |
| Promethazine | 1 | suppository | NA | NA | NA | 11.5 | 0.0 | 17.0 | 13.2 | 0.0 | 20.5 | 4.1 | 0.0 | 2.0 |
| Promethazine | 1 | syrup | 7.0 | 0.0 | 6.6 | 13.3 | 0.0 | 14.8 | 14.0 | 0.0 | 17.7 | 10.2 | 0.0 | 2.0 |
| Promethazine | 1 | tablet | 7.0 | 0.0 | 5.6 | 11.8 | 0.0 | 12.9 | 12.9 | 0.0 | 14.4 | 7.1 | 0.0 | 6.1 |
| Rolapitant, Varubi | 0 | tablet | 22.9 | 3.7 | 40.2 | 83.0 | 32.6 | 12.1 | 81.4 | 27.4 | 12.6 | 89.8 | 55.1 | 10.2 |
| Scopolamine | 1 | patch | 2.1 | 0.0 | 19.0 | 38.1 | 2.9 | 7.4 | 40.0 | 1.5 | 7.7 | 30.6 | 8.2 | 6.1 |

© 2025 Vu K et al. *JAMA Network Open*.

**eTable 4.** Number of Formulary Plans by State

| State | ACA | Medicaid |
|---|---|---|
| Alabama | 5 | 1 |
| Alaska | 2 | 1 |
| Arizona | 9 | 8 |
| Arkansas | 3 | 1 |
| California | 12 | 22 |
| Colorado | 6 | 4 |
| Connecticut | 2 | 0 |
| Delaware | 3 | 3 |
| District of Columbia | 0 | 4 |
| Florida | 14 | 9 |
| Georgia | 10 | 4 |
| Hawaii | 2 | 5 |
| Idaho | 6 | 1 |
| Illinois | 17 | 6 |
| Indiana | 4 | 5 |
| Iowa | 1 | 4 |
| Kansas | 7 | 3 |
| Kentucky | 4 | 7 |
| Louisiana | 7 | 7 |
| Maine | 3 | 1 |
| Maryland | 3 | 10 |
| Massachusetts | 1 | 5 |
| Michigan | 11 | 9 |
| Minnesota | 5 | 10 |
| Mississippi | 6 | 4 |

| State | ACA | Medicaid |
|---|---|---|
| Missouri | 11 | 2 |
| Montana | 4 | 1 |
| Nebraska | 2 | 3 |
| Nevada | 7 | 5 |
| New Hampshire | 3 | 4 |
| New Jersey | 6 | 6 |
| New Mexico | 4 | 4 |
| New York | 6 | 14 |
| North Carolina | 8 | 5 |
| North Dakota | 2 | 2 |
| Ohio | 11 | 10 |
| Oklahoma | 7 | 1 |
| Oregon | 4 | 9 |
| Pennsylvania | 8 | 8 |
| Rhode Island | 2 | 4 |
| South Carolina | 5 | 5 |
| South Dakota | 2 | 1 |
| Tennessee | 7 | 3 |
| Texas | 15 | 15 |
| Utah | 7 | 5 |
| Vermont | 1 | 1 |
| Virginia | 8 | 6 |
| Washington | 10 | 6 |
| West Virginia | 2 | 1 |
| Wisconsin | 15 | 2 |

© 2025 Vu K et al. *JAMA Network Open*.

**eTable 5.** Percentage of Covered Antiemetic Formulations Subject to Utilization Management in ACA Marketplace, by Plan

| Plan | PA (%) | ST(%) | QL(%) |
|---|---|---|---|
| Aetna Health Exchange Plan Banner Health | 8.55 | 0.00 | 35.95 |
| Aetna Health Exchange Plan California | 8.55 | 0.00 | 35.95 |
| Aetna Health Exchange Plan Delaware | 8.55 | 0.00 | 35.95 |
| Aetna Health Exchange Plan Florida | 8.55 | 0.00 | 35.95 |
| Aetna Health Exchange Plan Georgia | 8.55 | 0.00 | 35.95 |
| Aetna Health Exchange Plan Illinois | 8.55 | 0.00 | 35.95 |
| Aetna Health Exchange Plan Innovation Health | 8.55 | 0.00 | 35.95 |
| Aetna Health Exchange Plan Missouri | 8.55 | 0.00 | 35.95 |
| Aetna Health Exchange Plan Nevada | 8.55 | 0.00 | 35.95 |
| Aetna Health Exchange Plan New Jersey | 8.55 | 0.00 | 35.95 |
| Aetna Health Exchange Plan North Carolina | 8.55 | 0.00 | 35.95 |
| Aetna Health Exchange Plan Texas | 8.55 | 0.00 | 35.95 |
| Aetna Health Exchange Plan Virginia | 8.55 | 0.00 | 35.95 |
| Alliant Health Plans 3 Tier Formulary | 0.00 | 0.52 | 59.41 |
| Ambetter Absolute Total Care HIX | 0.86 | 0.00 | 58.38 |
| Ambetter Arizona Complete Health HIX | 2.94 | 0.00 | 58.38 |
| Ambetter IlliniCare HIX | 3.45 | 0.00 | 75.65 |
| Ambetter NH HIX | 0.35 | 0.00 | 48.19 |
| Ambetter PA Health and Wellness HIX | 0.86 | 0.00 | 58.38 |
| Ambetter from Buckeye Community Health Plan HIX OH | 3.45 | 0.00 | 57.34 |
| Ambetter from Coordinated Care HIX | 0.86 | 0.00 | 74.61 |
| Ambetter from Home State Health HIX | 0.86 | 0.00 | 58.38 |
| Ambetter from Louisiana Healthcare Connections HIX | 0.87 | 0.00 | 57.94 |
| Ambetter from MHS Indiana HIX | 3.45 | 0.00 | 57.34 |
| Ambetter from Magnolia Health Plan | 0.69 | 0.00 | 56.82 |
| Ambetter from Meridian MI HIX | 0.87 | 0.00 | 57.94 |
| Ambetter from Nebraska Total Care | 3.49 | 0.00 | 57.94 |
| Ambetter from Peach State Health Plan HIX | 3.45 | 0.00 | 75.65 |
| Ambetter from SilverSummit HealthPlan NV HIX | 0.86 | 0.00 | 58.38 |
| Ambetter from Sunflower Health Plan KS HIX | 0.86 | 0.00 | 58.38 |
| Ambetter from Sunshine Health HIX | 3.45 | 0.00 | 58.38 |
| Ambetter from Superior Health Plan HIX | 3.45 | 0.00 | 58.38 |
| Ambetter from WellCare of Kentucky HIX | 3.49 | 0.00 | 57.94 |
| Ambetter from WellCare of New Jersey HIX | 3.49 | 0.00 | 57.94 |
| Ambetter from Western Sky Community Care NM HIX | 3.49 | 0.00 | 57.94 |
| Ambetter of Alabama HIX | 0.87 | 0.00 | 57.94 |
| Ambetter of Arkansas HIX | 3.45 | 0.00 | 57.34 |
| Ambetter of North Carolina HIX | 3.45 | 0.00 | 58.38 |
| Ambetter of Oklahoma HIX | 3.49 | 0.00 | 57.94 |
| Ambetter of TN HIX | 0.86 | 0.00 | 58.38 |
| AmeriHealth Caritas Next DE HIX | 0.87 | 0.00 | 33.51 |
| AmeriHealth Caritas Next FL HIX | 0.87 | 0.00 | 33.51 |
| AmeriHealth Caritas Next NC HIX | 0.87 | 0.00 | 33.51 |
| AmeriHealth NJ HIX Value 3 Tier | 2.59 | 0.00 | 3.63 |
| Anthem BlueCross BlueShield HIX CO | 57.86 | 0.52 | 69.26 |
| Anthem BlueCross BlueShield HIX CT | 54.75 | 0.52 | 69.26 |
| Anthem BlueCross BlueShield HIX IN | 55.15 | 0.52 | 69.98 |
| Anthem BlueCross BlueShield HIX KY | 54.58 | 0.52 | 69.26 |
| Anthem BlueCross BlueShield HIX ME | 54.58 | 0.52 | 67.01 |
| Anthem BlueCross BlueShield HIX MO | 54.92 | 0.52 | 69.26 |
| Anthem BlueCross BlueShield HIX NH | 54.92 | 0.00 | 69.26 |
| Anthem BlueCross BlueShield HIX NV | 54.75 | 0.52 | 69.26 |
| Anthem BlueCross BlueShield HIX OH | 54.58 | 0.52 | 69.26 |
| Anthem BlueCross BlueShield HIX VA | 54.58 | 0.52 | 69.26 |
| Anthem BlueCross BlueShield HIX WI | 55.32 | 0.52 | 69.98 |
| Anthem BlueCross HIX CA | 58.03 | 0.52 | 69.26 |
| Arkansas BlueCross BlueShield HIX | 8.46 | 0.00 | 35.58 |
| Ascension HIX | 0.87 | 0.00 | 1.22 |
| Aspirus Arise HIX | 0.00 | 0.00 | 3.63 |
| AultCare MarketPlace HIX OH | 0.17 | 0.00 | 54.92 |
| Avera Health Plan HIX | 0.00 | 0.00 | 17.27 |
| BC of Idaho Qualified Health Plans | 0.00 | 0.00 | 32.82 |
| BCBS Kansas City Kansas HIX | 0.00 | 0.00 | 27.92 |
| BCBS Kansas City Missouri HIX | 0.00 | 0.00 | 27.92 |
| BCBS Michigan 5 Tier HIX | 0.35 | 0.35 | 33.33 |
| BCBS Tennessee HIX | 32.64 | 0.00 | 0.00 |
| BCBS Tennessee Prevent HIX | 32.64 | 0.00 | 0.00 |
| BCBS of AZ HIX EverydayHealth, TrueHealth and Essential | 0.52 | 0.17 | 63.73 |
| BCBS of AZ HIX Standardized Plans | 0.52 | 0.17 | 63.35 |
| BCBS of Alabama Source Plus Rx 1.0 Six Tier HIX | 0.00 | 0.52 | 35.06 |
| BCBS of Alabama SourceRx 1.0 Four Tier | 0.00 | 0.52 | 33.16 |
| BCBS of Arizona HIX Portfolio & SimpleHealth | 0.52 | 0.17 | 63.73 |
| BCBS of Georgia HIX GA | 54.75 | 0.52 | 69.26 |
| BCBS of Illinois Marketplace 5 Tier Generics Plus HIX | 0.00 | 0.00 | 33.68 |
| BCBS of Kansas BlueCare HIX | 0.00 | 0.00 | 0.00 |
| BCBS of Louisiana 2 Tier HIX | 0.52 | 0.00 | 68.39 |
| BCBS of Louisiana 3 Tier HIX | 0.52 | 0.00 | 68.39 |
| BCBS of Louisiana 4 Tier Covered Drug List | 0.52 | 0.00 | 69.11 |
| BCBS of Minnesota BasicRx MN HIX | 0.00 | 0.00 | 67.01 |
| BCBS of Montana Marketplace 4 Tier HIX | 0.00 | 0.00 | 58.64 |
| BCBS of Montana Marketplace Generics Plus HIX | 0.00 | 0.00 | 58.03 |
| BCBS of New Mexico HIX | 0.00 | 0.00 | 47.67 |
| BCBS of North Dakota HIX | 0.00 | 0.52 | 67.18 |
| BCBS of Oklahoma 4 Tier HIX | 0.00 | 0.00 | 58.64 |
| BCBS of Oklahoma Marketplace 5 Tier Generics Plus | 0.00 | 0.00 | 58.03 |
| BCBS of South Carolina BlueEssentials HIX | 0.00 | 0.00 | 25.04 |
| BCBS of Texas HIX 4 Tier | 0.00 | 0.00 | 58.64 |
| BCBS of Texas HIX 6 Tier | 0.00 | 0.00 | 55.44 |
| BCBS of Vermont HIX | 1.38 | 0.00 | 29.02 |
| BCBS of Wyoming BlueSelect 4 Tier HIX | 0.00 | 0.00 | 56.48 |
| BCN Michigan HIX MI | 0.35 | 0.35 | 33.33 |
| Blue Cross Blue Shield of Illinois Marketplace 4 Tier HIX | 0.00 | 0.00 | 58.64 |
| Blue Cross Blue Shield of Illinois Marketplace 4 Tier HMO-HIX | 0.00 | 0.00 | 58.64 |
| Blue Cross Blue Shield of Illinois Marketplace 6 Tier HMO-HIX | 0.00 | 0.00 | 40.93 |
| Blue Cross Blue Shield of Rhode Island HIX | 0.00 | 0.00 | 56.48 |
| Blue Shield HIX California | 0.35 | 0.17 | 35.41 |
| BridgeSpan Essential Formulary HIX OR and WA | 0.00 | 0.00 | 1.04 |
| CDPHP Formulary 2 HIX NY | 0.35 | 0.00 | 38.69 |
| CHRISTUS Health Plan Louisiana HIX | 0.00 | 0.00 | 69.95 |
| CHRISTUS Health Plan Texas HIX | 0.00 | 0.00 | 69.95 |
| Capital BlueCross Value | 2.59 | 0.17 | 57.51 |
| Capital Health Plan HIX | 0.00 | 0.00 | 68.06 |
| CareFirst Formulary 2 HIX | 8.46 | 0.52 | 35.75 |
| CareSource HIX GA | 2.59 | 0.00 | 57.51 |
| CareSource HIX NC | 2.62 | 0.00 | 58.12 |
| CareSource Just4Me HIX IN | 2.59 | 0.00 | 57.51 |
| CareSource Just4Me HIX KY | 2.59 | 0.00 | 57.51 |
| CareSource Just4Me HIX OH | 2.59 | 0.00 | 57.51 |
| CareSource Just4Me HIX WV | 2.59 | 0.00 | 57.51 |
| Childrens Community Health Plan HIX | 51.64 | 10.36 | 68.91 |
| Chinese Community Health Plan HIX | 0.00 | 0.00 | 3.45 |
| Cigna Health Plans VA HIX | 0.35 | 0.00 | 3.45 |
| Cigna Plus GA 5-Tier HIX | 0.35 | 0.00 | 3.49 |
| Cigna Plus MS 5-Tier HIX | 0.52 | 0.00 | 3.49 |
| Cigna Plus PA 5-Tier HIX | 0.52 | 0.00 | 3.49 |
| Cigna Plus TX 5-Tier HIX | 0.52 | 0.00 | 3.49 |
| Cigna Premier IN 5-Tier HIX | 0.52 | 0.00 | 3.49 |
| Cigna Premier SC 5-Tier HIX | 0.52 | 0.00 | 3.49 |
| Cigna Rx Essential 5-Tier HIX | 0.17 | 0.00 | 3.28 |
| Cigna Rx Essential UT 5-Tier HIX | 0.17 | 0.00 | 3.28 |
| Cigna Rx Plus HIX FL | 0.35 | 0.00 | 3.45 |
| Cigna Rx Plus HIX IL | 0.52 | 0.00 | 3.63 |
| Cigna Rx Plus HIX NC | 0.52 | 0.00 | 3.63 |
| Cigna Rx Plus HIX TN | 0.52 | 0.00 | 3.63 |
| Cigna Rx Premier 5 Tier AZ HIX | 0.52 | 0.00 | 3.63 |
| Common Ground Healthcare Cooperative HIX WI | 0.00 | 0.00 | 27.63 |
| Community First Health Plans | 0.86 | 0.86 | 45.94 |
| Community Health Choice HIX TX | 0.00 | 0.00 | 3.45 |
| Community Health Network of Washington Cascade Select HIX | 0.00 | 0.00 | 68.94 |
| CommunityCare OK HIX | 8.55 | 0.00 | 35.95 |
| ConnectiCare Freedom HIX CT | 0.00 | 0.00 | 69.95 |
| Cox Health MO HIX | 0.00 | 0.00 | 35.75 |
| Dean Health Plan HDHP HIX WI | 0.00 | 0.00 | 3.45 |
| Dean Health Plan HIX WI | 0.00 | 0.00 | 3.45 |
| Denver Health Elevate HIX CO | 0.00 | 0.00 | 6.22 |
| EmblemHealth Select Care HIX | 0.00 | 0.00 | 69.95 |
| Empire BlueCross HIX NY | 54.75 | 0.52 | 69.26 |
| First Choice Next SC HIX | 0.87 | 0.00 | 33.51 |
| Florida Blue Care Choices HIX | 0.86 | 0.17 | 65.29 |
| Florida Blue Care Choices HSA HIX | 0.86 | 0.17 | 41.97 |
| Florida Blue ValueScript Rx HIX | 0.35 | 0.00 | 0.35 |
| Florida Health Care Plan HIX | 3.97 | 0.17 | 30.92 |
| Geisinger Marketplace Formulary | 0.52 | 0.00 | 0.86 |
| Group Health Cooperative South Central Wisconsin HIX | 0.00 | 0.00 | 4.49 |
| Gundersen Lutheran Health Plan | 10.54 | 0.00 | 13.82 |
| HMO Partners, Inc. | 0.00 | 0.00 | 35.41 |
| HMSA Metallic Prescription Formulary | 0.00 | 0.17 | 0.52 |
| Harvard Pilgrim Core Formulary 5 Tier | 0.17 | 0.00 | 4.32 |
| Harvard Pilgrim Value Formulary 5 Tier | 0.17 | 0.00 | 4.49 |
| Health Alliance Medical Plans HIX | 0.00 | 0.00 | 3.97 |

© 2025 Vu K et al. *JAMA Network Open*.

| Plan | PA (%) | ST(%) | QL(%) |
|---|---|---|---|
| Health Alliance Plan 6 Tier Formulary | 0.00 | 0.00 | 39.38 |
| Health First Health Plans Florida HIX | 0.17 | 0.00 | 36.10 |
| Health Net of California Essential Rx HIX | 0.00 | 0.00 | 44.04 |
| Health New England 5 Tier HIX | 12.74 | 10.30 | 38.22 |
| Health Plan of Nevada 4 Tier HIX | 0.00 | 0.00 | 35.92 |
| HealthPartners MN Preferred Rx 3 Tier | 0.00 | 0.00 | 8.03 |
| HealthPartners MN Preferred Rx 4 Tier | 0.00 | 0.00 | 8.55 |
| HealthPartners WI Preferred Rx 4 Tier | 0.00 | 0.00 | 0.00 |
| HealthPartners WI Preferred Rx 5 Tier | 0.00 | 0.00 | 0.00 |
| Highmark BCBS Delaware HIX Comprehensive | 0.00 | 0.00 | 52.85 |
| Highmark Essential HIX | 0.00 | 0.00 | 52.85 |
| Hometown Health HIX | 0.00 | 0.00 | 6.81 |
| Horizon BlueCross BlueShield of NJ HIX | 0.52 | 0.52 | 65.80 |
| IdealCare from Sendero Health Plans | 0.00 | 0.00 | 3.63 |
| Imperial Health Plan HIX | 0.00 | 0.00 | 36.13 |
| Independence Blue Cross HIX 4 Tier | 2.59 | 0.00 | 3.63 |
| Independent Health HIX NY | 0.69 | 2.42 | 4.66 |
| Kaiser Permanente Colorado - Denver/Boulder/Northern HIX | 0.00 | 0.00 | 0.00 |
| Kaiser Permanente Georgia HIX | 0.00 | 36.44 | 11.92 |
| Kaiser Permanente Hawaii HIX 4 Tier | 0.00 | 0.00 | 0.00 |
| Kaiser Permanente Mid-Atlantic HIX | 0.00 | 0.00 | 0.00 |
| Kaiser Permanente Northern California HIX | 0.00 | 0.00 | 2.94 |
| Kaiser Permanente Oregon HIX | 0.17 | 0.00 | 2.76 |
| Kaiser Permanente Washington HIX | 0.00 | 0.00 | 0.00 |
| LA Care Marketplace HIX CA | 0.00 | 0.00 | 1.04 |
| LifeWise M4 Formulary HIX | 0.00 | 0.00 | 35.58 |
| MVP Health Care HIX NY | 0.00 | 0.00 | 3.97 |
| Maine Community Health Options | 0.00 | 0.00 | 69.95 |
| McLaren Health Plan HIX MI | 0.00 | 0.00 | 6.74 |
| Medica AZ, WI HIX | 0.70 | 0.52 | 68.94 |
| Medica IA, MN HIX | 0.70 | 0.52 | 68.94 |
| Medica KS, MO, NE, ND, OK HIX | 0.70 | 0.52 | 68.94 |
| Medical Mutual of OH High Performance Plus HIX | 0.00 | 0.17 | 68.39 |
| MercyCare Health Plans HIX WI | 0.00 | 0.00 | 4.49 |
| Moda Health Plan of Alaska HIX | 0.00 | 3.80 | 41.97 |
| Moda Health Plan of Idaho HIX | 0.00 | 3.84 | 41.36 |
| Moda Health Plan of Oregon HIX | 0.00 | 3.63 | 17.44 |
| Moda Health Plan of Texas HIX | 0.00 | 3.66 | 16.58 |
| Molina Healthcare of California HIX | 3.28 | 0.00 | 55.79 |
| Molina Healthcare of Florida HIX | 4.32 | 0.00 | 55.79 |
| Molina Healthcare of Idaho HIX | 3.32 | 0.00 | 55.32 |
| Molina Healthcare of Illinois HIX | 3.32 | 0.00 | 55.32 |
| Molina Healthcare of Kentucky HIX | 3.32 | 0.00 | 55.32 |
| Molina Healthcare of Michigan HIX | 3.28 | 0.00 | 55.79 |
| Molina Healthcare of Mississippi HIX | 3.28 | 0.00 | 55.79 |
| Molina Healthcare of New Mexico HIX | 3.28 | 0.00 | 55.79 |
| Molina Healthcare of Ohio HIX | 3.28 | 0.00 | 55.79 |
| Molina Healthcare of South Carolina HIX | 4.32 | 0.00 | 54.75 |
| Molina Healthcare of Texas HIX | 4.32 | 0.00 | 55.79 |
| Molina Healthcare of Utah HIX | 4.32 | 0.00 | 55.79 |

| Plan | PA (%) | ST(%) | QL(%) |
|---|---|---|---|
| Molina Healthcare of Washington HIX | 3.28 | 0.00 | 55.79 |
| Molina Healthcare of Wisconsin HIX | 3.28 | 0.00 | 55.79 |
| Mountain Health Coop HIX | 0.35 | 0.00 | 67.36 |
| MyPriority MI 5 Tier HIX | 0.00 | 0.17 | 3.28 |
| Neighborhood Health Plan 6T Formulary | 9.50 | 0.52 | 35.58 |
| Network Health Plan WI HIX | 0.00 | 3.28 | 68.22 |
| Optima Health Plan HIX VA | 22.97 | 0.52 | 43.87 |
| Oscar AZ HIX | 0.17 | 0.00 | 35.92 |
| Oscar FL HIX | 0.17 | 0.00 | 35.92 |
| Oscar GA HIX | 0.17 | 0.00 | 35.92 |
| Oscar HIX NY | 0.00 | 0.00 | 36.10 |
| Oscar IA HIX | 8.55 | 0.00 | 36.47 |
| Oscar IL HIX | 0.00 | 0.00 | 36.47 |
| Oscar KS HIX | 0.17 | 0.00 | 35.92 |
| Oscar MI HIX | 0.00 | 0.00 | 36.10 |
| Oscar MO HIX | 0.00 | 0.00 | 36.10 |
| Oscar NC HIX | 8.55 | 0.00 | 36.47 |
| Oscar NE HIX | 0.00 | 0.00 | 36.47 |
| Oscar NJ HIX | 0.17 | 0.00 | 35.92 |
| Oscar OH HIX | 0.17 | 0.00 | 35.92 |
| Oscar OK HIX | 0.17 | 0.00 | 36.30 |
| Oscar PA HIX | 0.00 | 0.00 | 36.10 |
| Oscar TN HIX | 0.00 | 0.00 | 36.10 |
| Oscar TX HIX | 0.17 | 0.00 | 35.92 |
| Oscar VA HIX | 8.46 | 0.00 | 36.10 |
| PacificSource HIX ID | 0.35 | 0.00 | 11.57 |
| PacificSource HIX MT | 0.35 | 0.00 | 11.57 |
| PacificSource HIX OR | 0.35 | 0.00 | 17.10 |
| PacificSource HIX WA | 0.35 | 0.00 | 11.57 |
| Paramount Individual Exchange and Essential (HIX OH) | 8.46 | 0.00 | 35.58 |
| Physicians Health Plan of Michigan HIX | 0.35 | 0.00 | 3.45 |
| Premera HIX M2 | 0.00 | 0.00 | 35.58 |
| Premera HIX M4 | 0.00 | 0.00 | 35.58 |
| Presbyterian Health Plan HIX | 3.66 | 0.00 | 66.32 |
| Prevea360 Health Plan HDHP HIX | 0.00 | 0.00 | 3.45 |
| Prevea360 Health Plan HIX | 0.00 | 0.00 | 3.45 |
| Providence Health Plan Formulary M | 0.00 | 0.00 | 3.66 |
| Providence Health Plan Formulary N | 0.00 | 0.00 | 3.66 |
| Regence Six-Tier Drug List - Core | 0.00 | 0.00 | 1.04 |
| Rocky Mountain Essential Plus HIX | 0.00 | 0.00 | 36.30 |
| Rocky Mountain Option HIX | 0.00 | 0.00 | 36.47 |
| Sanford Health Plan HIX | 0.00 | 0.00 | 3.97 |
| Scott and White Health Plan Essential Health Benefits Formulary | 0.52 | 0.52 | 27.92 |
| Security Health Plan of Wisconsin HIX | 0.00 | 0.00 | 69.95 |
| SelectHealth RxCore Idaho HIX | 0.00 | 0.00 | 50.09 |
| SelectHealth RxCore Nevada HIX | 0.00 | 0.00 | 50.26 |
| SelectHealth RxCore Utah HIX | 0.00 | 0.00 | 52.68 |
| SelectHealth RxCore Utah HIX 4 Tier | 0.00 | 0.00 | 58.64 |
| Sharp Health Plan Three Tier | 0.00 | 0.00 | 35.41 |
| Sierra Health and Life Four Tier | 0.00 | 0.00 | 0.00 |
| St. Luke's Health Plan HIX | 0.00 | 0.00 | 0.00 |
| SummaCare HIX OH | 3.28 | 0.00 | 38.51 |

| Plan | PA (%) | ST(%) | QL(%) |
|---|---|---|---|
| Sutter Health | 0.00 | 0.00 | 0.00 |
| The Health Plan Upper Ohio Valley Incentive Formulary | 0.00 | 0.00 | 35.58 |
| UCare Choice HIX MN | 0.00 | 0.00 | 34.72 |
| UPMC Advantage Choice | 51.64 | 10.36 | 68.91 |
| UnitedHealthcare 4T AL HIX | 0.00 | 0.00 | 36.47 |
| UnitedHealthcare 4T AZ HIX | 0.00 | 0.00 | 36.47 |
| UnitedHealthcare 4T FL HIX | 0.00 | 0.00 | 36.47 |
| UnitedHealthcare 4T GA HIX | 0.00 | 0.00 | 36.47 |
| UnitedHealthcare 4T IL HIX | 0.00 | 0.00 | 36.47 |
| UnitedHealthcare 4T KS HIX | 0.00 | 0.00 | 36.47 |
| UnitedHealthcare 4T LA HIX | 0.00 | 0.00 | 36.47 |
| UnitedHealthcare 4T MI HIX | 0.00 | 0.00 | 36.47 |
| UnitedHealthcare 4T MO HIX | 0.00 | 0.00 | 36.47 |
| UnitedHealthcare 4T MS HIX | 0.00 | 0.00 | 36.47 |
| UnitedHealthcare 4T NC HIX | 0.00 | 0.00 | 36.47 |
| UnitedHealthcare 4T OH HIX | 0.00 | 0.00 | 36.47 |
| UnitedHealthcare 4T OK HIX | 0.00 | 0.00 | 36.47 |
| UnitedHealthcare 4T TN HIX | 0.00 | 0.00 | 36.47 |
| UnitedHealthcare 4T TX HIX | 0.00 | 0.00 | 36.47 |
| UnitedHealthcare 4T VA HIX | 0.00 | 0.00 | 36.47 |
| UnitedHealthcare AL HIX | 0.00 | 0.00 | 36.47 |
| UnitedHealthcare AZ HIX | 0.00 | 0.00 | 36.47 |
| UnitedHealthcare FL HIX | 0.00 | 0.00 | 36.47 |
| UnitedHealthcare GA HIX | 0.00 | 0.00 | 36.47 |
| UnitedHealthcare IL HIX | 0.00 | 0.00 | 36.47 |
| UnitedHealthcare KS HIX | 0.00 | 0.00 | 36.47 |
| UnitedHealthcare LA HIX | 0.00 | 0.00 | 36.47 |
| UnitedHealthcare MD HIX | 0.00 | 0.00 | 36.47 |
| UnitedHealthcare MI HIX | 0.00 | 0.00 | 36.47 |
| UnitedHealthcare MO HIX | 0.00 | 0.00 | 36.47 |
| UnitedHealthcare MS HIX | 0.00 | 0.00 | 36.47 |
| UnitedHealthcare NC HIX | 0.00 | 0.00 | 36.47 |
| UnitedHealthcare OH HIX | 0.00 | 0.00 | 36.47 |
| UnitedHealthcare OK HIX | 0.00 | 0.00 | 36.47 |
| UnitedHealthcare Oxford Health Plan Advantage Four Tier | 0.52 | 0.52 | 3.28 |
| UnitedHealthcare TN HIX | 0.00 | 0.00 | 36.47 |
| UnitedHealthcare TX HIX | 0.00 | 0.00 | 36.47 |
| UnitedHealthcare VA HIX | 0.00 | 0.00 | 36.47 |
| UnitedHealthcare WA HIX | 0.00 | 0.00 | 36.47 |
| Unity Health Plan Standard Four Tier | 0.00 | 0.00 | 14.85 |
| University of Utah HIX | 0.35 | 0.52 | 67.36 |
| Valley Health Plan HIX CA | 0.00 | 0.00 | 0.52 |
| Vantage Health Plan LA | 3.45 | 0.00 | 68.39 |
| WellFirst Health HDHP HIX IL | 0.00 | 0.00 | 3.66 |
| WellFirst Health HMO HIX IL | 0.00 | 0.00 | 3.66 |
| WellFirst Health Plan HDHP HIX MO | 0.00 | 0.00 | 3.63 |
| WellFirst Health Plan HIX MO | 0.00 | 0.00 | 3.63 |
| Wellmark Blue Rx Essentials | 0.00 | 0.00 | 2.59 |
| Western Health Advantage HIX CA | 0.69 | 0.00 | 35.75 |

© 2025 Vu K et al. *JAMA Network Open*.

**eTable 6.** Percentage of Covered Antiemetic Formulations Subject to Utilization Management in Medicaid Marketplace, by Plan

| Plan | PA (%) | ST(%) | QL(%) |
|---|---|---|---|
| Absolute Total Care | 2.27 | 1.21 | 64.95 |
| Aetna Better Health Texas STAR | 71.45 | 0.91 | 0.00 |
| Aetna Better Health of IL | 3.03 | 0.45 | 79.39 |
| Aetna Better Health of KanCare | 0.76 | 0.00 | 0.30 |
| Aetna Better Health of Kentucky | 33.08 | 1.06 | 33.84 |
| Aetna Better Health of Louisiana | 7.25 | 1.21 | 0.91 |
| Aetna Better Health of Maryland | 2.72 | 0.45 | 63.29 |
| Aetna Better Health of Michigan | 4.83 | 0.45 | 31.27 |
| Aetna Better Health of New Jersey | 3.02 | 0.60 | 79.00 |
| Aetna Better Health of Virginia | 3.32 | 0.76 | 28.70 |
| Alabama State Medicaid | 31.12 | 0.60 | 46.07 |
| Alameda Alliance for Health Medi-Cal | 0.60 | 0.00 | 3.02 |
| Alaska State Medicaid | 3.02 | 0.00 | 0.00 |
| AllCare (OHP) | 4.08 | 0.00 | 1.21 |
| Aloha Care Quest | 3.78 | 0.00 | 98.49 |
| AmeriHealth Caritas Delaware Medicaid | 27.64 | 0.76 | 1.96 |
| AmeriHealth Caritas District of Columbia | 5.89 | 0.15 | 38.52 |
| AmeriHealth Caritas Louisiana | 7.25 | 1.21 | 0.91 |
| AmeriHealth Caritas New Hampshire | 1.96 | 0.60 | 61.93 |
| AmeriHealth Caritas North Carolina Medicaid | 50.92 | 0.61 | 30.43 |
| AmeriHealth Caritas Ohio | 13.00 | 0.61 | 20.34 |
| AmeriHealth Caritas Pennsylvania | 15.11 | 0.15 | 80.97 |
| Amerigroup Community Care Georgia | 15.86 | 0.30 | 98.49 |
| Amerigroup Community Care Louisiana | 7.25 | 1.21 | 0.91 |
| Amerigroup Community Care Maryland | 6.95 | 0.76 | 82.63 |
| Amerigroup Community Care Nevada | 14.65 | 0.15 | 98.49 |
| Amerigroup Community Care New Jersey | 15.86 | 0.15 | 98.49 |
| Amerigroup Community Care Tennessee | 78.55 | 6.65 | 61.18 |
| Amerigroup District of Columbia | 14.83 | 0.00 | 99.69 |
| Amerigroup Iowa Health Link | 15.71 | 11.48 | 56.65 |
| Amerigroup Texas STAR | 71.45 | 0.91 | 0.00 |
| Amerigroup Washington Apple Health | 3.32 | 0.60 | 1.51 |
| Amida Care | 33.08 | 29.61 | 12.84 |
| Anthem Blue Cross and Blue Shield Ohio | 13.00 | 0.61 | 20.34 |
| Anthem BlueCross BlueShield Kentucky Medicaid | 33.08 | 0.15 | 33.84 |
| Anthem BlueCross California Medicaid | 0.76 | 0.15 | 3.02 |
| Anthem Healthkeepers Plus | 7.25 | 0.15 | 48.94 |
| Anthem Indiana Hoosier Healthwise | 49.85 | 0.00 | 97.13 |
| Arizona Complete Health-Complete Care Plan | 3.02 | 0.45 | 61.33 |
| Arizona State Medicaid Fee For Service | 74.92 | 0.45 | 58.61 |
| Arkansas Medicaid | 77.95 | 0.45 | 45.77 |
| BCBS ND Medicaid | 8.87 | 0.15 | 47.71 |
| BCBS New Mexico Centennial Care | 33.38 | 0.00 | 59.52 |
| BCBS Western New York Medicaid | 34.29 | 29.76 | 12.84 |
| BCBS of Minnesota Medicaid GenRx | 48.19 | 0.00 | 39.43 |
| BMC Mass Health | 49.55 | 30.51 | 33.84 |
| Blue Cross Community ICP | 40.48 | 0.00 | 62.08 |
| Blue Cross Complete | 3.63 | 0.30 | 33.08 |
| Blue Cross and Blue Shield of Texas STAR | 71.45 | 0.91 | 0.00 |
| BlueChoice Health Plan of South Carolina Medicaid | 15.86 | 0.00 | 98.64 |
| BlueCross BlueShield of Tennessee TennCare | 78.55 | 6.65 | 61.18 |
| Buckeye Community Health Plan | 12.99 | 1.36 | 20.09 |
| CHP+ offered by Colorado Access | 11.63 | 0.00 | 2.72 |
| CalOptima Medi-Cal | 1.21 | 0.00 | 3.93 |
| CalViva Health Medi-Cal | 1.81 | 0.15 | 3.02 |
| Care 1st Health Plan Medi-Cal | 3.02 | 0.00 | 3.02 |
| Care 1st HealthPlan Arizona | 2.42 | 0.60 | 61.33 |
| CareOregon (OHP) | 21.30 | 0.00 | 5.59 |
| CareSource Georgia Medicaid | 14.35 | 0.00 | 19.64 |
| CareSource HIP Basic | 4.83 | 0.30 | 75.23 |
| CareSource Ohio Medicaid | 33.23 | 0.60 | 20.09 |
| Carolina Complete Health | 5.96 | 3.06 | 0.00 |
| Cascade Comprehensive Care HMO Medicaid | 3.78 | 0.00 | 25.98 |
| CenCal Health | 2.11 | 0.00 | 3.02 |
| Colorado State Medicaid | 4.98 | 1.21 | 30.21 |
| Community Health Choice STAR | 71.45 | 0.91 | 0.00 |
| Community Health Group Medi-Cal | 2.11 | 0.00 | 3.02 |
| Community Health Plan of Imperial Valley Medi-Cal | 0.61 | 0.00 | 3.06 |
| Community Health Plan of Washington Apple Health | 3.17 | 0.45 | 0.00 |
| Contra Costa Health Plan Medi-Cal | 2.42 | 0.00 | 3.02 |
| Cook Children's Health Plan STAR | 71.45 | 0.91 | 0.00 |
| Coordinated Care | 4.83 | 1.21 | 32.48 |
| CountyCare Health Plan | 3.02 | 0.00 | 33.53 |
| Coventry Florida MMA | 5.14 | 0.00 | 18.58 |
| CoventryCares of West Virginia | 32.78 | 4.38 | 28.55 |
| Delaware Medical Assistance Program(DMAP) | 33.23 | 0.76 | 0.00 |
| Denver Health Medicaid Choice & CHP+ | 2.72 | 0.00 | 25.98 |
| Driscoll Children's Health Plan STAR | 71.45 | 0.91 | 0.00 |
| Empire BlueCross BlueShield HealthPlus | 34.29 | 29.76 | 12.84 |
| Excellus Child Health Plus | 1.96 | 1.06 | 4.23 |
| Fidelis Care New York | 33.08 | 29.61 | 12.84 |
| FirstCare STAR | 71.45 | 0.91 | 0.00 |
| Florida Medicaid | 5.14 | 0.00 | 18.58 |
| Geisinger Health Plan Family | 53.78 | 0.00 | 80.06 |
| Georgia Department of Community Health | 2.57 | 0.30 | 58.01 |
| Gold Coast Health Plan | 0.76 | 0.00 | 3.02 |
| HAP CareSource MI Formulary | 3.67 | 0.00 | 34.25 |
| HIP Medicaid/FHPlus | 33.08 | 29.61 | 12.84 |
| HMSA Quest | 2.57 | 0.00 | 28.25 |
| Health Choice Arizona | 46.98 | 0.45 | 52.27 |
| Health Choice Utah | 33.69 | 0.91 | 61.78 |
| Health Net Medi-Cal | 1.66 | 0.15 | 3.02 |
| Health Partners of Philadelphia | 70.85 | 0.15 | 80.97 |
| Health Plan of Nevada Medicaid | 15.26 | 0.91 | 98.49 |
| Health Plan of San Joaquin | 2.87 | 0.00 | 3.02 |
| Health Plan of San Mateo | 0.91 | 0.00 | 3.02 |
| HealthPartners Minnesota Health Care Programs | 31.42 | 0.45 | 0.15 |
| Healthfirst NY Medicaid Managed Care | 33.08 | 29.61 | 12.84 |
| Healthy Blue MO HealthNet | 49.01 | 2.29 | 28.86 |
| Healthy Blue North Carolina Medicaid | 5.96 | 3.06 | 0.00 |
| Healthy U | 32.48 | 0.91 | 61.93 |
| Hennepin Health Plan | 11.33 | 0.60 | 30.36 |
| Highmark Health Options | 15.56 | 9.67 | 48.34 |
| Highmark WholeCare Pennsylvania | 14.07 | 0.00 | 47.55 |
| Horizon NJ Health | 4.08 | 0.15 | 59.97 |
| Humana CareSource Kentucky Medicaid | 33.08 | 0.76 | 33.84 |
| Humana Florida MMA | 5.14 | 0.00 | 18.58 |
| Humana Healthy Horizons Louisiana | 6.12 | 0.46 | 0.92 |
| Humana Healthy Horizons Ohio | 13.00 | 0.61 | 20.34 |
| Humana Healthy Horizons South Carolina Medicaid | 13.61 | 0.00 | 51.22 |
| IMCare | 2.72 | 0.60 | 2.57 |
| Idaho Medicaid | 3.93 | 2.57 | 0.00 |
| Illinois Medicaid | 3.63 | 0.45 | 0.00 |
| Independent Health MediSource and CHIP | 32.93 | 29.61 | 12.84 |
| Indiana Medicaid | 1.66 | 0.30 | 77.34 |
| Inland Empire Health Plan Medi-Cal | 1.96 | 0.30 | 3.02 |
| InterCommunity Health Network CCO | 4.68 | 0.00 | 28.25 |
| Iowa State Medicaid | 15.71 | 11.48 | 56.65 |
| Iowa Total Care Formulary | 15.71 | 11.48 | 56.65 |
| Jai Medical Systems | 5.74 | 0.45 | 58.61 |
| Kaiser Permanente Hawaii QUEST | 7.10 | 0.00 | 0.00 |
| Kaiser Permanente Maryland HealthChoice | 2.87 | 0.45 | 30.36 |
| Kaiser Permanente Medi-Cal Managed Care | 1.06 | 0.00 | 3.02 |
| Kentucky Health Choices Medicaid | 32.93 | 0.45 | 33.84 |
| Kern Family Health Care Medi-Cal | 1.51 | 0.00 | 3.02 |
| Keystone First | 15.11 | 0.15 | 80.97 |
| LA Care Health Plan Medi-Cal | 1.96 | 0.00 | 3.02 |
| Louisiana Healthcare Connections | 7.25 | 1.21 | 0.91 |
| Louisiana Medicaid | 7.25 | 1.21 | 0.91 |
| MDwise HIP Basic Plan | 3.02 | 0.15 | 78.55 |
| MHS Healthy Indiana HIP Basic | 49.40 | 46.68 | 1.96 |
| MO Healthnet (formerly Missouri Medicaid) | 48.64 | 2.27 | 28.55 |
| MVP Option and Option Family | 33.08 | 29.61 | 12.84 |
| Magellan Complete Care of Arizona | 3.93 | 0.45 | 61.33 |
| Magellan Complete Care of Virginia | 6.04 | 0.00 | 29.00 |
| Magnolia Health Plan | 18.28 | 0.30 | 21.45 |
| MaineCare | 5.74 | 5.29 | 30.06 |
| Maryland Physicians Care | 6.95 | 0.45 | 58.61 |
| Maryland State Medicaid | 6.04 | 0.45 | 35.80 |
| Masshealth Medicaid | 50.76 | 31.42 | 33.84 |
| McLaren Health Plan Medicaid | 4.83 | 0.45 | 33.53 |
| MedStar DC Healthy Families | 11.18 | 0.00 | 4.23 |
| MedStar Family Choice Health Plan | 5.89 | 0.45 | 34.14 |
| Medi Cal | 1.81 | 0.00 | 3.02 |
| Medica Minnesota Medicaid | 11.32 | 0.00 | 62.39 |
| Medical Assistance (Pennsylvania Medicaid) | 69.03 | 10.73 | 79.15 |
| Medical Assistance District of Columbia | 24.02 | 0.00 | 0.00 |
| Mercy Care Health Plan BadgerCare Plus | 28.40 | 0.30 | 61.03 |
| Mercy Care Plan | 3.78 | 0.45 | 63.29 |
| Meridian Health Plan of Illinois | 5.14 | 0.00 | 25.98 |

© 2025 Vu K et al. *JAMA Network Open*.

| Plan | PA (%) | ST(%) | QL(%) |
|---|---|---|---|
| Meridian Health Plan of Michigan | 4.68 | 0.30 | 33.23 |
| MetroPlus Health Plan New York Medicaid | 33.08 | 29.61 | 12.84 |
| Michigan Medicaid | 3.78 | 0.00 | 31.57 |
| Minnesota Medicaid | 32.63 | 0.60 | 28.55 |
| Mississippi Medicaid | 18.28 | 0.30 | 21.45 |
| Moda Health - Oregon Health Plan | 1.66 | 0.15 | 29.61 |
| Molina HealthCare of Utah Medicaid | 37.46 | 1.36 | 74.17 |
| Molina Healthcare Iowa Health Link | 14.68 | 11.32 | 57.34 |
| Molina Healthcare Texas STAR | 71.45 | 0.91 | 0.00 |
| Molina Healthcare of Florida MMA | 5.14 | 0.00 | 18.58 |
| Molina Healthcare of Illinois | 3.32 | 0.00 | 81.72 |
| Molina Healthcare of Michigan | 2.72 | 0.30 | 32.63 |
| Molina Healthcare of MississippiCAN | 18.28 | 0.30 | 21.45 |
| Molina Healthcare of Nevada Medicaid | 4.59 | 0.00 | 79.82 |
| Molina Healthcare of Ohio | 33.23 | 0.76 | 20.09 |
| Molina Healthcare of South Carolina | 5.74 | 19.64 | 52.42 |
| Molina Healthcare of WA | 3.78 | 0.00 | 48.49 |
| Molina Medi-Cal | 1.66 | 0.00 | 3.02 |
| Montana Medicaid | 14.35 | 0.60 | 29.15 |
| Nebraska State Medicaid | 2.11 | 0.76 | 47.13 |
| Nebraska Total Care | 47.73 | 0.45 | 29.15 |
| Neighborhood Health Plan MassHealth | 49.85 | 30.97 | 33.84 |
| Neighborhood Health Plan of RI Rite Care | 6.95 | 0.30 | 0.00 |
| Nevada State Medicaid | 30.82 | 0.60 | 28.40 |
| New Hampshire Healthy Families | 1.21 | 2.72 | 32.48 |
| New Hampshire State Medicaid | 0.30 | 2.27 | 31.87 |
| New Jersey State Medicaid | 0.00 | 0.00 | 0.00 |
| New Mexico Medicaid | 0.00 | 0.00 | 0.00 |
| New York State Medicaid | 33.08 | 29.76 | 12.84 |
| North Carolina Medicaid | 7.25 | 4.08 | 0.00 |
| North Dakota State Medicaid | 8.76 | 0.15 | 48.34 |
| Ohana QUEST Integration | 5.59 | 0.45 | 0.30 |
| Ohio Medicaid | 12.99 | 0.76 | 20.09 |
| Oklahoma Medicaid (SoonerCare) | 4.98 | 3.47 | 33.38 |
| Optima Family Care/FAMIS | 2.27 | 1.21 | 0.00 |
| Oregon Health Plan (Medicaid) | 1.96 | 0.76 | 0.45 |
| PacificSource Community Solutions | 0.91 | 3.32 | 3.17 |
| Parkland HEALTHfirst | 71.45 | 0.91 | 0.00 |
| Partnership HealthPlan of CA Medi-Cal | 3.02 | 0.00 | 3.02 |
| Passport Health Plan | 32.02 | 0.45 | 33.84 |
| Peach State Health Plan Medicaid | 3.02 | 1.21 | 63.29 |
| Presbyterian Health Plan Centennial Care | 5.74 | 0.30 | 57.86 |
| Prestige Health Choice MMA | 5.14 | 0.00 | 18.58 |
| PrimeWest Health Medicaid | 11.63 | 0.45 | 35.95 |
| Priority Health Michigan Medicaid | 3.93 | 0.45 | 33.53 |
| Priority Partners | 2.87 | 0.45 | 60.57 |
| Providence Health Plan-Oregon Health Plan | 0.91 | 0.00 | 2.27 |
| Rhode Island Medicaid | 14.80 | 9.97 | 0.00 |
| RightCare from Scott & White | 67.98 | 0.91 | 0.00 |
| Riverside Health | 5.44 | 0.45 | 58.61 |
| Rocky Mountain Health Plans Prime Medicaid and CHP Plus | 14.65 | 0.00 | 98.34 |
| San Francisco Health Plan Medi-Cal | 1.66 | 0.00 | 3.02 |
| Santa Clara Family Health Plan Medi-Cal | 1.96 | 0.00 | 3.02 |
| SelectHealth Community Care | 36.10 | 0.91 | 58.91 |
| Seton Health Plan STAR | 71.45 | 0.91 | 0.00 |
| SilverSummit HealthPlan Nevada Medicaid | 2.57 | 0.76 | 69.34 |

| Plan | PA (%) | ST(%) | QL(%) |
|---|---|---|---|
| Simply Healthcare MMA | 5.14 | 0.00 | 18.58 |
| South Carolina Medicaid | 11.33 | 0.45 | 0.00 |
| South Country Health Alliance | 12.84 | 0.45 | 35.05 |
| South Dakota Medicaid | 46.68 | 0.00 | 0.30 |
| South Florida Community Care Network MMA | 5.14 | 0.00 | 18.58 |
| Sunflower State Health Plan KANCARE | 0.76 | 0.00 | 0.30 |
| Sunshine State Health Plan MMA Specialty | 5.14 | 0.00 | 18.58 |
| Superior HealthPlan STAR | 71.45 | 0.91 | 0.00 |
| Texas Childrens Health Plan STAR | 71.45 | 0.91 | 0.00 |
| Texas Medicaid | 71.45 | 0.00 | 0.00 |
| The Health Plan West Virginia Medicaid | 32.78 | 4.38 | 28.55 |
| Total Care | 33.99 | 29.61 | 12.84 |
| Trillium Medicaid (OHP) | 1.96 | 0.15 | 28.25 |
| Tufts Health RITogether | 3.02 | 0.45 | 59.82 |
| Tufts Health Together | 49.09 | 30.51 | 33.84 |
| UCare Minnesota Medicaid | 11.93 | 0.00 | 22.66 |
| UPMC For You | 54.68 | 0.15 | 80.97 |
| UniCare West Virginia Medicaid | 32.78 | 4.38 | 28.55 |
| UnitedHealthCare Community Plan Kentucky | 32.37 | 0.46 | 34.20 |
| UnitedHealthcare Community Plan - New York | 34.29 | 29.61 | 12.84 |
| UnitedHealthcare Community Plan Arizona Physicians IPA | 50.00 | 0.00 | 99.40 |
| UnitedHealthcare Community Plan MPlus MMA | 5.14 | 0.00 | 18.58 |
| UnitedHealthcare Community Plan MississippiCAN | 18.28 | 0.30 | 21.45 |
| UnitedHealthcare Community Plan QUEST Integration Program | 22.66 | 0.45 | 61.93 |
| UnitedHealthcare Community Plan TennCare | 78.55 | 6.65 | 61.18 |
| UnitedHealthcare Community Plan of Kansas - KANCARE | 0.76 | 0.00 | 0.30 |
| UnitedHealthcare Community Plan of Louisiana | 7.25 | 1.21 | 0.91 |
| UnitedHealthcare Community Plan of MN Medicaid | 13.00 | 0.00 | 100.00 |
| UnitedHealthcare Community Plan of Maryland | 6.34 | 0.45 | 84.14 |
| UnitedHealthcare Community Plan of Michigan (formerly Great Lakes HP) | 4.68 | 0.45 | 32.63 |
| UnitedHealthcare Community Plan of Nebraska | 12.54 | 0.30 | 95.47 |
| UnitedHealthcare Community Plan of New Jersey | 16.01 | 0.00 | 99.40 |
| UnitedHealthcare Community Plan of North Carolina | 5.96 | 3.06 | 0.00 |
| UnitedHealthcare Community Plan of Ohio | 33.23 | 0.45 | 21.30 |
| UnitedHealthcare Community Plan of Pennsylvania | 70.85 | 0.30 | 80.97 |
| UnitedHealthcare Community Plan of Rhode Island | 12.84 | 0.00 | 59.06 |
| UnitedHealthcare Community Plan of Texas STAR | 71.45 | 0.91 | 0.00 |
| UnitedHealthcare Community Plan of Virginia Medallion 4.0 | 5.59 | 0.00 | 68.13 |
| UnitedHealthcare Community Plan of Washington Apple Health | 3.78 | 0.45 | 55.89 |
| University Family Care | 74.92 | 0.45 | 58.61 |
| Upper Peninsula Health Plan | 4.83 | 0.45 | 33.23 |
| Utah Medicaid | 34.29 | 1.21 | 30.06 |
| VNSNY CHOICE SelectHealth Formulary | 33.08 | 29.61 | 12.84 |
| Vermont Medicaid | 13.75 | 3.32 | 47.43 |
| Virginia Medicaid | 25.38 | 4.38 | 0.00 |
| Washington State Medicaid | 3.02 | 0.76 | 1.51 |
| Well Sense Health Plan | 14.20 | 0.76 | 60.57 |
| WellCare of Kentucky | 32.02 | 0.00 | 33.84 |
| WellCare of New Jersey | 1.66 | 0.00 | 19.94 |
| West Virginia Medicaid | 32.78 | 4.38 | 28.55 |
| Western Oregon Advanced Health CCO Medicaid | 1.66 | 0.00 | 25.98 |
| Western Sky Community Care Centennial Care | 3.47 | 0.60 | 69.34 |
| Wisconsin Medicaid | 28.40 | 0.30 | 61.03 |
| Yamhill Community Care | 0.91 | 0.00 | 2.27 |

© 2025 Vu K et al. *JAMA Network Open*.

**eTable 7.** Formulary Tier Distribution for Antiemetic Drugs

| | | ACA | | Medicaid | |
|---|---|---|---|---|---|
| Brand Name | Tier | N | Percentage | N | Percentage |
| Akynzeo | 1 | 80 | 26.58 | 6 | 2.27 |
| Akynzeo | 2 | 76 | 25.25 | 238 | 90.15 |
| Akynzeo | 5 | 1 | 0.33 | 0 | 0.00 |
| Akynzeo | 7 | 94 | 31.23 | 1 | 0.38 |
| Akynzeo | 8 | 27 | 8.97 | 10 | 3.79 |
| Akynzeo | 9 | 3 | 1.00 | 9 | 3.41 |
| Akynzeo | 10 | 1 | 0.33 | 0 | 0.00 |
| Akynzeo | 11 | 19 | 6.31 | 0 | 0.00 |
| Aprepitant | 2 | 54 | 1.00 | 240 | 5.05 |
| Aprepitant | 3 | 2658 | 49.06 | 39 | 0.82 |
| Aprepitant | 4 | 360 | 6.64 | 1752 | 36.87 |
| Aprepitant | 5 | 1731 | 31.95 | 2274 | 47.85 |
| Aprepitant | 6 | 474 | 8.75 | 417 | 8.78 |
| Aprepitant | 7 | 126 | 2.33 | 30 | 0.63 |
| Aprepitant | 11 | 15 | 0.28 | 0 | 0.00 |
| Emend | 1 | 121 | 13.40 | 81 | 10.23 |
| Emend | 2 | 155 | 17.17 | 571 | 72.10 |
| Emend | 4 | 3 | 0.33 | 0 | 0.00 |
| Emend | 5 | 0 | 0.00 | 2 | 0.25 |
| Emend | 7 | 540 | 59.80 | 4 | 0.51 |
| Emend | 8 | 65 | 7.20 | 115 | 14.52 |
| Emend | 9 | 4 | 0.44 | 19 | 2.40 |
| Emend | 11 | 15 | 1.66 | 0 | 0.00 |
| Haloperidol | 2 | 143 | 0.44 | 1063 | 3.87 |
| Haloperidol | 3 | 3816 | 11.85 | 14 | 0.05 |
| Haloperidol | 4 | 13 | 0.04 | 35 | 0.13 |
| Haloperidol | 5 | 23825 | 73.97 | 21671 | 78.93 |
| Haloperidol | 6 | 1614 | 5.01 | 4673 | 17.02 |
| Haloperidol | 7 | 2702 | 8.39 | 0 | 0.00 |
| Haloperidol | 11 | 94 | 0.29 | 0 | 0.00 |
| Metoclopramide Hydrochloride | 2 | 0 | 0.00 | 46 | 4.53 |
| Metoclopramide Hydrochloride | 3 | 29 | 9.63 | 4 | 0.39 |
| Metoclopramide Hydrochloride | 4 | 0 | 0.00 | 134 | 13.19 |
| Metoclopramide Hydrochloride | 5 | 246 | 81.73 | 490 | 48.23 |
| Metoclopramide Hydrochloride | 6 | 1 | 0.33 | 12 | 1.18 |
| Metoclopramide Hydrochloride | 7 | 24 | 7.97 | 0 | 0.00 |
| Metoclopramide Hydrochloride | 11 | 1 | 0.33 | 193 | 19.00 |
| Olanzapine | 2 | 885 | 1.56 | 205 | 0.41 |
| Olanzapine | 3 | 12542 | 22.05 | 731 | 1.47 |
| Olanzapine | 4 | 2693 | 4.73 | 4627 | 9.27 |
| Olanzapine | 5 | 37913 | 66.64 | 34327 | 68.80 |
| Olanzapine | 6 | 1205 | 2.12 | 10006 | 20.05 |
| Olanzapine | 7 | 1521 | 2.67 | 0 | 0.00 |
| Olanzapine | 11 | 130 | 0.23 | 0 | 0.00 |
| Ondansetron | 2 | 187 | 0.33 | 15 | 0.03 |
| Ondansetron | 3 | 10568 | 18.78 | 15 | 0.03 |
| Ondansetron | 4 | 15 | 0.03 | 150 | 0.30 |
| Ondansetron | 5 | 45176 | 80.26 | 46292 | 93.77 |
| Ondansetron | 6 | 0 | 0.00 | 2881 | 5.84 |
| Ondansetron | 7 | 169 | 0.30 | 15 | 0.03 |
| Ondansetron | 11 | 172 | 0.31 | 0 | 0.00 |
| Prochlorperazine | 2 | 28 | 4.65 | 28 | 5.30 |
| Prochlorperazine | 3 | 234 | 38.87 | 4 | 0.76 |
| Prochlorperazine | 4 | 2 | 0.33 | 108 | 20.45 |
| Prochlorperazine | 5 | 264 | 43.85 | 360 | 68.18 |
| Prochlorperazine | 7 | 72 | 11.96 | 28 | 5.30 |
| Prochlorperazine | 11 | 2 | 0.33 | 0 | 0.00 |
| Promethazine Hydrochloride | 2 | 3 | 0.02 | 518 | 1.49 |
| Promethazine Hydrochloride | 3 | 1477 | 10.01 | 328 | 0.94 |
| Promethazine Hydrochloride | 4 | 106 | 0.72 | 692 | 1.99 |
| Promethazine Hydrochloride | 5 | 10951 | 74.25 | 30201 | 86.66 |
| Promethazine Hydrochloride | 6 | 931 | 6.31 | 3096 | 8.88 |
| Promethazine Hydrochloride | 7 | 1240 | 8.41 | 13 | 0.04 |
| Promethazine Hydrochloride | 11 | 41 | 0.28 | 0 | 0.00 |
| Reglan | 1 | 101 | 16.78 | 98 | 18.56 |
| Reglan | 2 | 82 | 13.62 | 415 | 78.60 |
| Reglan | 4 | 2 | 0.33 | 0 | 0.00 |
| Reglan | 7 | 417 | 69.27 | 8 | 1.52 |
| Reglan | 8 | 0 | 0.00 | 3 | 0.57 |
| Reglan | 9 | 0 | 0.00 | 4 | 0.76 |
| Scopolamine | 2 | 396 | 34.92 | 126 | 8.61 |
| Scopolamine | 4 | 180 | 15.87 | 330 | 22.54 |
| Scopolamine | 6 | 0 | 0.00 | 30 | 2.05 |
| Scopolamine | 11 | 546 | 48.15 | 600 | 40.98 |
| Scopolamine | 12 | 12 | 1.06 | 378 | 25.82 |
| Varubi | 1 | 40 | 13.29 | 8 | 3.03 |
| Varubi | 2 | 74 | 24.58 | 218 | 82.58 |
| Varubi | 4 | 1 | 0.33 | 0 | 0.00 |
| Varubi | 7 | 50 | 16.61 | 23 | 8.71 |
| Varubi | 8 | 115 | 38.21 | 8 | 3.02 |
| Varubi | 9 | 15 | 4.98 | 7 | 2.65 |
| Varubi | 11 | 6 | 1.99 | 0 | 0.00 |
| Zyprexa | 1 | 620 | 15.84 | 641 | 18.68 |
| Zyprexa | 2 | 557 | 14.23 | 2342 | 68.24 |
| Zyprexa | 4 | 13 | 0.33 | 0 | 0.00 |
| Zyprexa | 7 | 2596 | 66.34 | 21 | 0.61 |
| Zyprexa | 8 | 73 | 1.87 | 284 | 8.28 |
| Zyprexa | 9 | 0 | 0.00 | 144 | 4.20 |
| Zyprexa | 10 | 21 | 0.54 | 0 | 0.00 |
| Zyprexa | 11 | 33 | 0.84 | 0 | 0.00 |

© 2025 Vu K et al. *JAMA Network Open*.

**eFigure 1**. Percentage of Covered Antiemetic Formulations Subject to Utilization Management, Medicaid MCO vs State

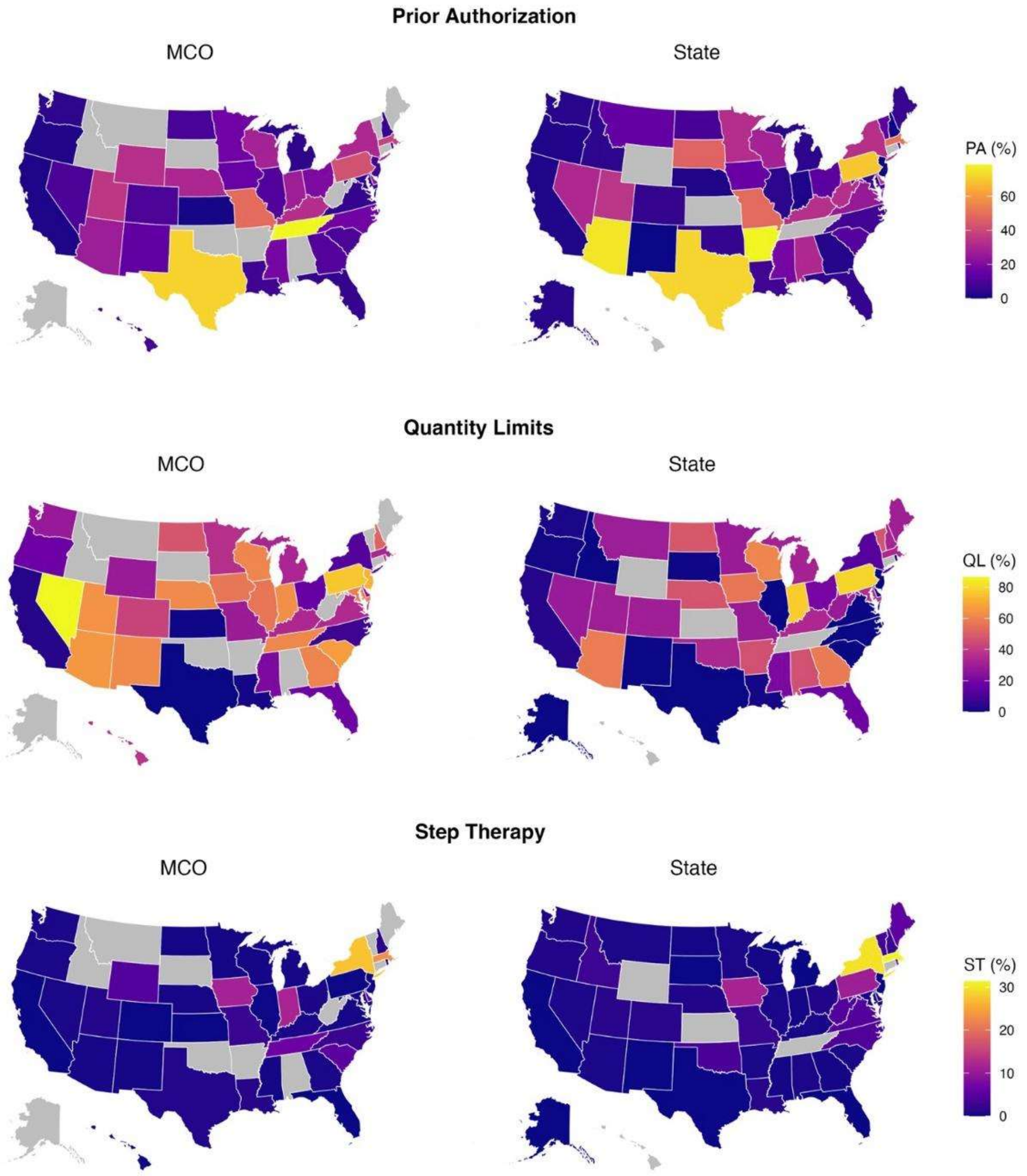


© 2025 Vu K et al. *JAMA Network Open*.

**eFigure 2.** Percentage of Covered Antiemetic Formulations Subject to Step Therapy, by Drug and Coverage Type

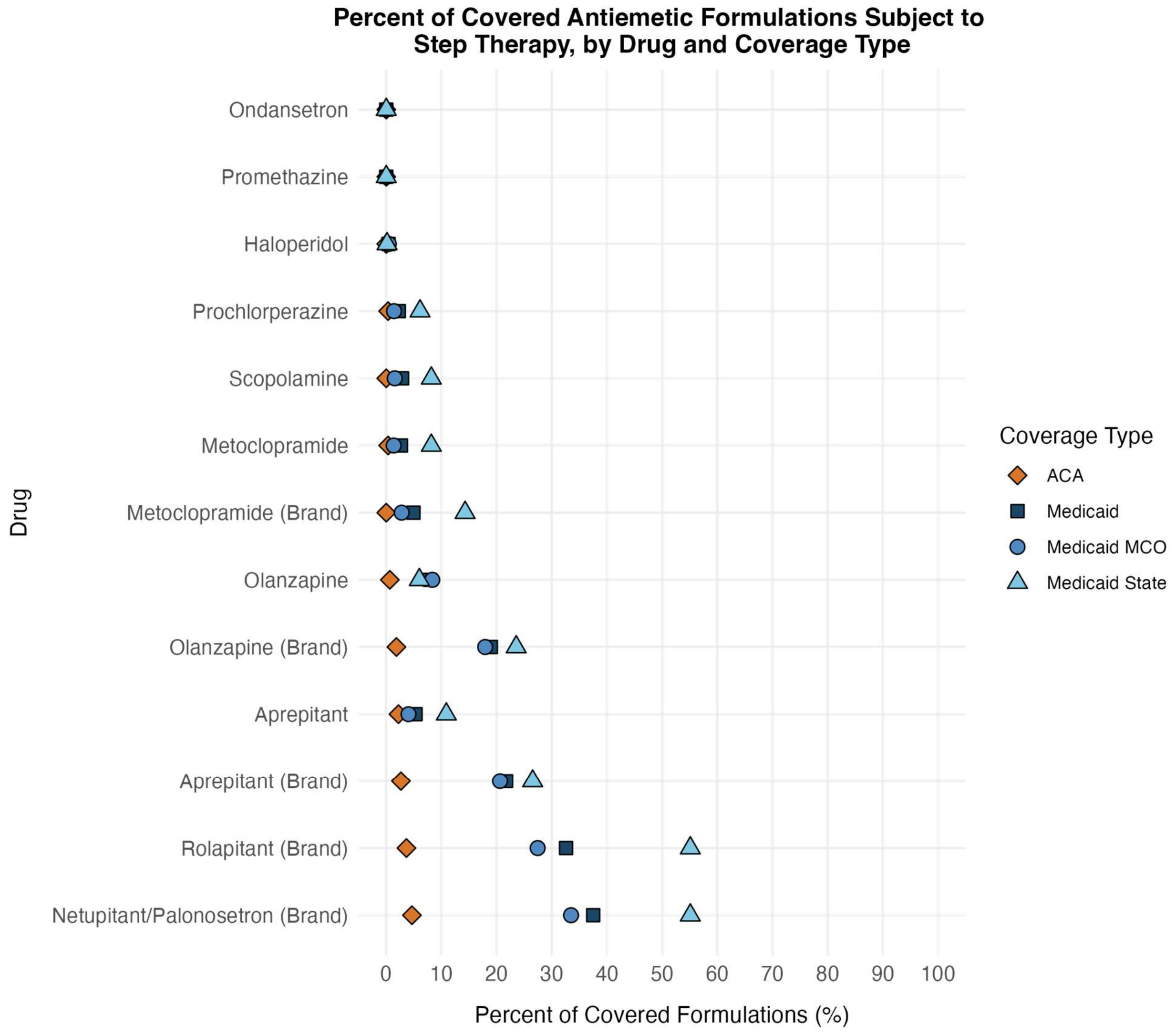


© 2025 Vu K et al. *JAMA Network Open*.
