# Supplementary material for: Prior Authorization, Quantity Limits, and Step Therapy for Patient-Administered Antiemetics
Source: JAMA Netw Open. 2025 Oct 6;8(10):e2535707. doi: 10.1001/jamanetworkopen.2025.35707 (PMC12501812; doi:10.1001/jamanetworkopen.2025.35707)
Supplement: Supplement 2. — Data Sharing Statement [file jamanetwopen-e2535707-s002.pdf]

Data Sharing Statement

Vu. Prior Authorization, Quantity Limits, and Step Therapy for Patient-Administered Antiemetics. *JAMA Netw Open*. Published October 06, 2025.
doi:10.1001/jamanetworkopen.2025.35707

Data
Data available: No

Additional Information
Explanation for why data not available: Our data use agreement with prevents us from sharing the formulary dataset used in this analysis. However, plans make formularies publicly available and can be looked up individually.
